# Revealing the role of regulatory microglial IRF7-NLRP3 interactions in optic nerve damage of normal-tension glaucoma based on single-cell RNA sequencing

**DOI:** 10.3389/fimmu.2025.1700998

**Published:** 2026-01-12

**Authors:** Leyi Qiu, Fengyi Guo, Xinna Liu, Di Zhang, Qi Wang, Mengxian Du, Qianmei Yuan, Shiqi Zhang, Wulian Song, Huiping Yuan

**Affiliations:** 1 Department of Ophthalmology, The Second Affiliated Hospital of Harbin Medical University, Harbin, China; 2 Harbin Medical University Key Laboratory of Myocardial Ischemia Ministry of Education, Harbin, China; 3 Future Medical Laboratory, The Second Affiliated Hospital of Harbin Medical University, Harbin, China

**Keywords:** OPTN (E50K) mutation, normal-tension glaucoma, microglia, optic nerve damage, single-cell RNA sequencing, IRF7, NLRP3

## Abstract

Normal-tension glaucoma (NTG) is a subtype of primary open-angle glaucoma (POAG). Patients with NTG still experience significant optic nerve damage despite maintaining normal intraocular pressures. The mechanism of optic nerve damage in glaucoma with normal pressure is still unclear. Research has shown that OPTN (E50K) mutations exacerbate the inflammatory response of retinal microglia. However, there is still a lack of evidence on how OPTN (E50K) mutations directly regulate their inflammatory pathways through key molecules. This study explores the role of microglial inflammation caused by the interaction between IRF7 and NLRP3 molecules in NTG optic nerve injury. Single-cell RNA sequencing (scRNA-seq) was employed to analyze retinal microglial cells from both wild-type (WT) and OPTN (E50K) mutant mice. The analysis revealed significant enrichment of inflammatory pathways and a critical role of IRF7 in modulating NLRP3 activation. Techniques such as Western blot (WB), qPCR, immunofluorescence (IF), and molecular docking were utilized to confirm the interactions between IRF7 and NLRP3. The findings demonstrate that the OPTN (E50K) mutation reduces the suppressive effect of IRF7 on NLRP3, leading to a pro-inflammatory microglial phenotype and exacerbating the optic nerve damage of NTG. This study provides a new therapeutic target for the treatment of NTG optic nerve damage.

## 1 Introduction

Glaucoma is a leading cause of irreversible vision loss worldwide and is primarily characterized by the apoptosis of retinal ganglion cells (RGCs). Normal tension glaucoma (NTG) is a subtype of primary open-angle glaucoma (POAG) (1) and is particularly common in Asian populations. A recent meta-analysis reported that up to 70% of Chinese POAG patients are diagnosed with NTG. Despite having normal intraocular pressure, NTG patients often suffer from progressive optic nerve damage, and conventional intraocular pressure-lowering therapies are often insufficient to prevent disease progression (2). The precise mechanisms underlying optic nerve damage in NTG remain largely unclear. Therefore, elucidating the pathological pathways leading to RGC damage in NTG is crucial for the development of novel therapeutic strategies aimed at delaying progressive optic nerve damage.

Following the identification of OPTN (E50K) mutation as a major causative gene for familial NTG in 2002 (3), we developed a corresponding mutant mouse model using CRISPR/Cas9 technology (4), which has been included in the laboratory animal database by the Jackson Laboratory, a leading biomedical research institution in the United States. Using this NTG mouse model, our research group has focused on elucidating the mechanisms and exploring potential treatments for optic nerve damage in NTG.

Building on this model, we performed proteomic profiling on retinal tissues from aged OPTN (E50K) and WT mice. The analysis revealed that the aged E50K retina exhibited a significant enrichment of microglia-associated proteins compared with WT controls, consistent with our previously published findings (5). We also observed an increased number of Iba-1^+^ microglia in the retinas of aged E50K mutant mice, particularly within the ganglion cell layer (GCL), indicating that the OPTN (E50K) mutation leads to microglial activation (5). Previous research has demonstrated that microglial activation not only heightens the sensitivity of RGCs to inflammatory damage but also ultimately results in optic nerve damage (6). Microglial activation is an early and critical event in glaucomatous optic neuropathy, with both protective and detrimental effects depending on disease stage and context (7). Notably, activation of the NLRP3 inflammasome in microglia has been implicated in RGC damage and is now regarded as a key driver of neuroinflammation in glaucoma (8). Our prior study also suggests that the OPTN (E50K) mutant mice and E50K-R28 cells exhibit robust activation of the NLRP3 inflammasome, characterized by increased expression of NLRP3, Caspase-1, IL-1β, and IL-18, accompanied by a pronounced retinal inflammatory cascade (9). This activation promotes the transformation of microglia into a pro-inflammatory phenotype, leading to the release of cytokines. These cytokines exacerbate RGC damage and apoptosis.

To precisely characterize molecular changes in microglia and investigate how the E50K mutation regulates their inflammatory pathways, we employed methods such as single-cell RNA sequencing (scRNA-seq), bioinformatics analysis, and differential gene expression profiling, and analyzed retinal microglial cells from both wild-type (WT) and OPTN (E50K) mutant mice. The results indicated that in aged E50K mutant mice, the type I interferon signaling pathway was significantly down-regulated, and regulatory factors related to the type I interferon signaling pathway, especially Interferon regulatory factor 7 (IRF7), were markedly down-regulated. In models of stroke, IRF4 and IRF5 were shown to direct pro- and anti-inflammatory microglial phenotypes, respectively, underscoring the broader role of the IRF family in neuroinflammation control (10). Furthermore, analysis using the STRING database confirmed an interaction between IRF7 and Trim30a, which is also downregulated and has been shown to exert a negative regulatory effect on NLRP3 expression. Based on these preliminary findings, we propose that the OPTN (E50K) mutation influences NLRP3 expression through IRF7, a key regulator of the type I interferon signaling pathway, thereby triggering the inflammatory activation of microglia and subsequent damage to RGCs. Together, our study systematically defines the IRF7-NLRP3 axis in OPTN (E50K) mutant microglia, offering novel mechanistic insight into inflammation-driven RGC damage in NTG.

## 2 Materials and methods

### 2.1 OPTN (E50K) mutation mice

All animal experiments were approved by the Ethics Committee of Harbin Medical University. OPTN (E50K) knock-in mice were generated as a normal-tension glaucoma (NTG) mouse model on a C57BL/6J background using CRISPR/Cas9 gene-editing technology, as previously reported (4). All experimental animals were 18- month- old OPTN (E50K) mice, and age-matched wild-type (WT) C57BL/6J as controls, exhibiting normal ocular and physiological conditions. Both male and female mice were included, and all experiments were performed using both eyes. All animals were housed in a specific pathogen-free (SPF) facility under a 12 h light/12 h dark cycle, with ad libitum access to food and water.

This mouse model exhibits normal intraocular pressure and age-related retinal ganglion cell degeneration, making it suitable for investigating NTG pathology.

### 2.2 Cell culture and transfection

The murine microglial BV2 cell line (AW-CNM081, Abiowell, China) was used in this study. Cells were cultured in RPMI 1640 medium supplemented with 10% fetal bovine serum (FBS) and 1% penicillin-streptomycin-amphotericin B and maintained at 37°C in a humidified incubator with 5% CO_2_. Lentiviral transfection was performed according to the manufacturer’s protocol to introduce the E50K mutation, resulting in the stable establishment of an E50K mutant BV2 cell line.

### 2.3 Light/dark transition test (L/D-T Test)

**Figure 1:**
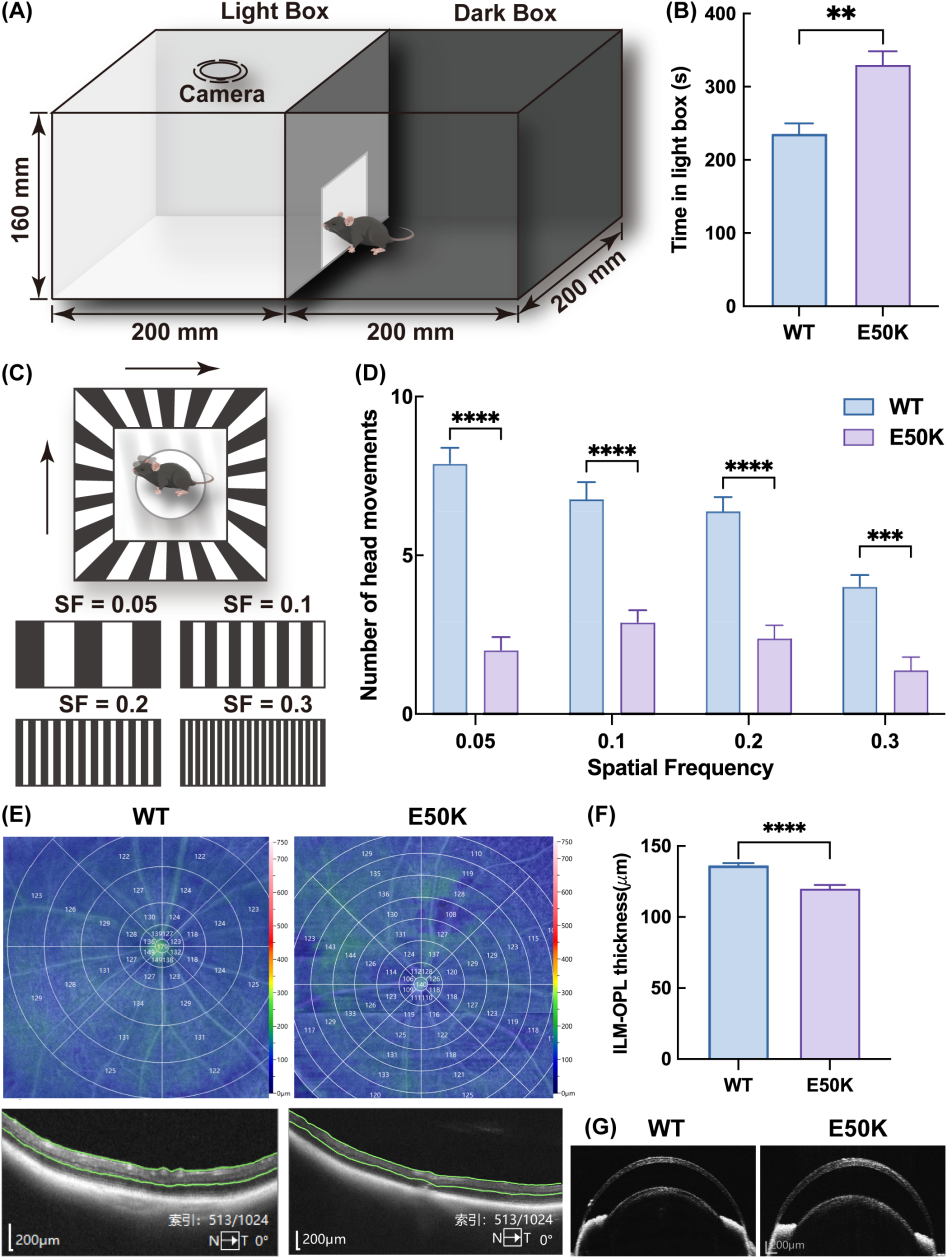
Analysis of visual function and retinal structure in aged E50K mutant mice and aged WT mice. **(A)** Light/Dark Transition Test Setup; **(B)** Time Spent in the Light Box: Bar graph illustrating time mice spent in the Light Box; n=7 **(C)** Optomotor Response Test Setup; **(D)** Optomotor Response Test Results: Bar graph displaying the average response at different spatial frequencies; n=8 **(E)** Retinal thickness map; **(F)** Bar graph of retinal thickness statistics. (Data are presented as mean ± SEM); n≥5 **(G)** Anterior segment OCTA images. **P < 0.01; ***P < 0.001; ****P < 0.0001.

To assess visual function in mice, a light/dark transition test was performed. The experimental device consisted of two connected chambers: a light box with a light source and a completely dark box. Mice were allowed to move freely between the chambers. After a 2-hour adaptation period in the dark environment, the mice were placed in the dark chamber for a 10-minute test (**Figure 1A**), and the time spent in the light chamber was recorded. This test primarily evaluates the visual sensitivity of mice, with prolonged stays in the light chamber, which may indicate impaired vision.

### 2.4 Optomotor response (OMR Test)

The optomotor response test evaluates the sensitivity of mice to visual stimuli at various spatial frequencies. The apparatus features an LED screen on the side wall displaying black and white striped gratings at different frequencies. Following a light adaptation period, the mice were positioned on a central platform, and their head movements were monitored by a top camera to assess their tracking behavior in response to the visual stimuli. Four spatial frequencies were tested (0.05, 0.1, 0.2, and 0.3 cycles per degree (CPD)), with the grating rotating clockwise at 12 degrees/s. The test lasted for 3 minutes (**Figure 1C**), and the head tracking movements were recorded and analyzed using automated intelligent software, complemented by video review. This test primarily evaluates visual responsiveness, particularly at higher spatial frequencies.

### 2.5 Optical coherence tomography angiography

Using Optical Coherence Tomography Angiography (OCTA, Toward Pi BM-400K, Beiming Kun), imaging of the retina and anterior segment was conducted on aged (18-month-old) E50K mutant mice and WT mice. In the area surrounding the central macula, retinal thickness was measured with precision. Automated software was employed to calculate retinal thickness for each region, spanning from the inner limiting membrane (ILM) to the outer plexiform layer (OPL). High-resolution scans of the anterior segment were obtained, providing clear visualization of structural details in the mice anterior segment. Consistent technical parameters were maintained throughout all scans to ensure reproducibility and accuracy across all measurements.

### 2.6 Single-cell RNA sequencing

Retinal tissues were rinsed in PBS and dissociated to remove non-retinal tissues, yielding a single-cell suspension. The cell concentration was adjusted to 700–1200 cells/µl, and scRNA-seq libraries were prepared using the 10 × Chromium system and sequenced on an Illumina NovaSeq 6000 platform at Lianchuan Biotech Co., Ltd., Hangzhou, China. Raw data were aligned, and gene expression matrices were quantified using CellRanger (v7.0.0). The resulting data were imported into Seurat (v4.1.0) for normalization (Normalization was performed using the LogNormalize method), cell filtering, subpopulation classification, and differential gene analysis followed by PCA dimensionality reduction and t-SNE (t-Distributed stochastic Neighbor Embedding) visualization. Differentially expressed genes were analyzed using the FindAllMarkers function, and GO enrichment analysis was performed using ggplot2, with parameters set as follows: ① genes expressed in more than 10% of cells in the target or control subgroup; ② p<0.05; ③ the threshold for gene expression multiples log2FC was 0.26.

### 2.7 Immunofluorescence

Cell slides were fixed with 4% paraformaldehyde (PFA) for 15 min at room temperature, washed with PBS, permeabilized with 0.5% Triton X-100 for 30 min, blocked with 5% BSA for 30 min, and incubated overnight at 4°C with primary antibodies: IRF7 (1:100, R381201, Positive Energy) and NLRP3 (1:100, 68102-1-Ig, Proteintech). The next day, cells were incubated at room temperature for 1 h with FITC-labelled goat anti-rabbit IgG (1:100, ZF-0311, ZSGB-BIO) and TRITC-labelled goat anti-mouse IgG (1:100, ZF-0313, ZSGB-BIO). Nuclei were counterstained with an anti-fluorescence quenching sealant containing DAPI (P0131, Beyotime, China). Two-channel imaging was performed using a fluorescence microscope (Leica Microsystems), and three-channel imaging using a confocal microscope (Carl Zeiss, LSM980). Fluorescence intensity was analyzed using ImageJ software, and Pearson’s r and Overlap_R were calculated using the JACoP plug-in to analyze colocalization.

### 2.8 Western blot

Retina tissue or cells were collected and lysed in RIPA buffer (P0013B, Beyotime) containing PMSF (ST506, Beyotime). After sonication, lysates were incubated on ice for 30 min and centrifuged at 12,000 rpm for 10 min at 4°C. Supernatants were collected, and protein concentrations were measured using a BCA kit (P0010, Beyotime). Samples were boiled at 100°C for 5 min, separated by 10% SDS-PAGE, and transferred to PVDF membranes. Membranes were blocked at room temperature with either QuickBlock™ solution (P0252, Beyotime) for 30 min or 5% skim milk for 1 h. Samples were then incubated overnight at 4°C with the following primary antibodies: IRF7 (1:1000, R381201, Positive Energy), NLRP3 (1:2000, 68102-1-Ig, Proteintech), OPTN (1:1000, 10837-1-AP, Proteintech), IL-1β (1:1000, 26048-1-AP, Proteintech), IL-18 (1:1000, 516737, Positive Energy), and β-actin (1:20000, 66009-1-Ig, Proteintech). The next day, after rinsing with an appropriate amount of TBST, membranes were incubated with horseradish peroxidase-conjugated goat anti-mouse IgG (ZB-2305, ZSGB-BIO) and goat anti-rabbit IgG (ZB-2301, ZSGB-BIO) at room temperature for 1 h. Protein bands were observed using a SuperSignal ECL Ultra-Sensitive Substrate (MA0186, Meilunbio^®^), quantified using ImageJ software, and the protein gray value was normalized using β-actin as an internal reference.

### 2.9 Real-time quantitative PCR

Total RNA was extracted from the retina and cells using the SteadyPure Quick TNA Extraction Kit (AG21023, AGBio). cDNA was synthesized using the NovoScript^®^ Plus All-in-one 1st Strand cDNA Synthesis SuperMix (gDNA Purge) (E047, Novoprotein) according to the manufacturer’s instructions. Then, qPCR was performed using NovoStart^®^ SYBR qPCR SuperMix Plus (E096, Novoprotein) as per the manufacturer’s protocol. Results were normalized to β-actin, and the ΔCt value was calculated. The relative expression of mRNA in each group was calculated using 2^-ΔΔCt^. The 2^-ΔΔCt^ method was applied to calculate relative gene expression levels between groups.

### 2.10 Co-immunoprecipitation

Cells were collected and lysed with an appropriate amount of cell lysis buffer. The mixture was well mixed and incubated on ice for 30 minutes, inverting every 10 minutes. After centrifugation at 12,000 rpm at 4 °C for 10 minutes, the supernatant was collected. 4-6 µl of primary antibody IRF7 (R381201, CUSABIO) and rabbit IgG (A7016, Beyotime) were added to Protein A/G Magnetic Beads (PB101-01, Vazyme), mixed, and incubated at 4°C for 2 h, separated by magnetic force, and the magnetic beads were collected and washed with an appropriate amount of PBST. The antigen sample was added to the antibody-magnetic bead complex, mixed well, and incubated at 4°C for 2 h under gentle rotation. The magnetic beads were separated by a magnet, the supernatant was discarded and washed three times with PBST. The magnetic beads were separated, the supernatant discarded, and 40 μl 2x Loading Buffer was added to the magnetic beads, mixed well, heated in a 95°C metal bath for 5 minutes, and the protein sample was prepared. Subsequently, the WB method was used to detect whether the two proteins interact with each other.

### 2.11 Molecular docking analysis

Structural data for IRF7 and NLRP3 proteins were obtained from the UniProt database, and the online Zdock website (https://zdock.wenglab.org/) was used to evaluate the interaction. The Top 10 Model was analyzed using the PDBePISA tool on the EMBL-EBI website (https://www.ebi.ac.uk/pdbe/pisa/). PDBePISA provided detailed parameters such as the change in binding interface free energy (Δ^i^G), interface area, hydrogen bonds, and salt bridges. Upon entering the structural data of IRF7 and NLRP3 into the platform, the binding interface characteristics were automatically calculated, with Δ^i^G assessing the stability of the complex and interface area serving as a supplementary indicator of binding strength. Hydrogen bonds and salt bridges at the binding interface were shown to stabilize the complex through non-covalent interactions. The PyMOL (v3.1.4) software was used to visualize the interface structure, displaying the interaction sites and their spatial distribution.

### 2.12 Statistical analysis

Analysis was conducted using GraphPad Prism software (v10.2.1). All data are expressed as mean ± SEM. Independent sample t-tests were employed to analyze significant differences between two groups of data, and one-way ANOVA was used to compare and test differences between multiple groups. P-value<0.05 was considered to be significantly different and statistically significant.

For all experiments involving repeated measurements, both biological and technical replicates were performed at least three times, including repeated measurements from the same sample (technical replicates) and independent repetitions using separately cultured cells or different biological individuals (biological replicates). All experiments were conducted with a minimum of three replicates.

## 3 Results

### 3.1 The effect of OPTN (E50K) mutation on mice’s visual function and retinal structure

The visual function of the mice was assessed using the light/dark transition (L/D-T) behavior test and the optokinetic response (OMR). The results from the L/D-T test revealed that 18-month-old E50K mutant mice spent significantly more time in the light chamber compared to WT mice, indicating that the E50K mutation leads to pronounced visual impairment and decreased visual sensitivity (**Figures 1A, B**). Furthermore, the OMR experiment demonstrated that the head movement frequency of 18-month-old E50K mutant mice across various spatial frequencies was markedly lower than that of WT mice. This result suggests that the E50K mutation not only reduces visual sensitivity but also impairs the ability to respond to complex visual stimuli, particularly at higher spatial frequencies (**Figures 1C, D**). These results confirm the visual dysfunction in OPTN (E50K) mutant mice. Additionally, optical coherence tomography angiography (OCTA) revealed that while the anterior segment structure of 18-month-old E50K mutant mice appears normal, their retinal thickness is significantly reduced (**Figures 1E-G**). Previous studies by our research group have verified that intraocular pressure in E50K mutant mice remains within the normal range (9). These findings further support the utility of the E50K mutant mice as a reliable model for studying the pathogenesis and developing treatment strategies for NTG.

### 3.2 Single-cell RNA sequencing identifies retinal cell subtypes

**Figure 2:**
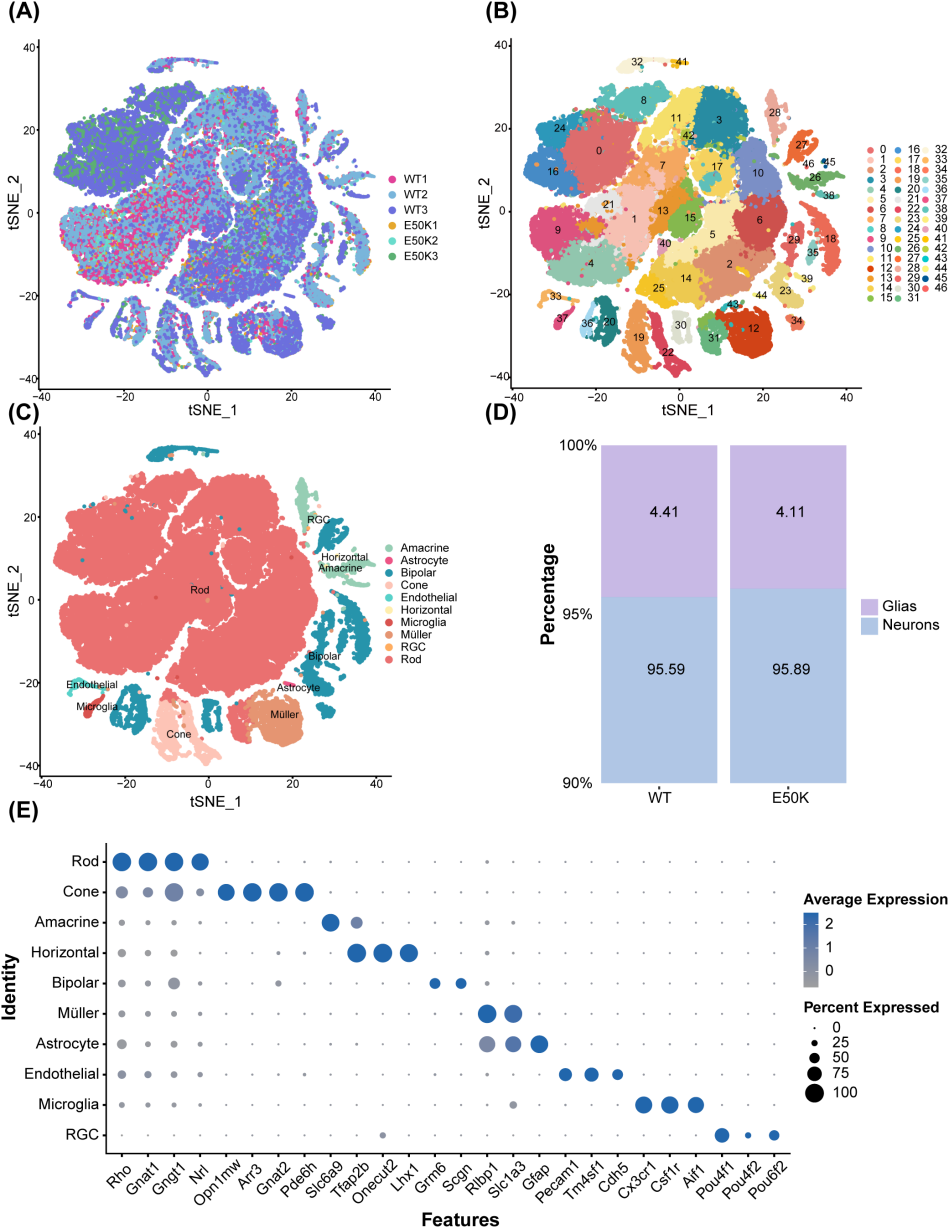
Comprehensive analysis of retinal cell types and gene expression in a single-cell sequencing study. **(A)** Single-cell distribution by sample origin; **(B)** Cell clustering of retinal tissue from E50K mutant and WT mice; **(C)** t-SNE plot displaying the classification of cells into ten types based on marker gene expression; **(D)** Proportions of neurons and glial cells in the retinas of E50K mutant and WT mice; **(E)** Dotplot showing the expression levels of primary marker genes for each cell subtype. n=3.

Retinal tissue samples from 18-month-old E50K mutant and age-matched WT mice were processed using scRNA-seq, resulting in the identification of 63,059 cells (**Figure 2A**). Based on gene expression profiles, these cells were classified into 46 distinct subpopulations, or clusters (**Figure 2B**).

By examining marker gene expression, the cells were categorized into ten major cell types: amacrine, astrocyte, bipolar, cone, endothelial, horizontal, microglia, Müller, RGC, and rod cells. The expression levels of key marker genes for each cell type are illustrated in a dotplot diagram (**Figure 2E**), while the t-SNE clustering diagram depicts the spatial distribution of these cell types (**Figure 2C**).

The analysis revealed that the proportions of neurons and glial cells in the retinas of 18-month-old E50K mutant and WT mice were comparable. Neurons accounted for 95.59% and 95.89% of total cells in the E50K mutant and WT groups, respectively, while glial cells represented 4.41% and 4.11% of the total cells in these groups (**Figure 2D**).

### 3.3 Microglial activation in inflammatory pathway modulation in NTG retina

**Figure 3:**
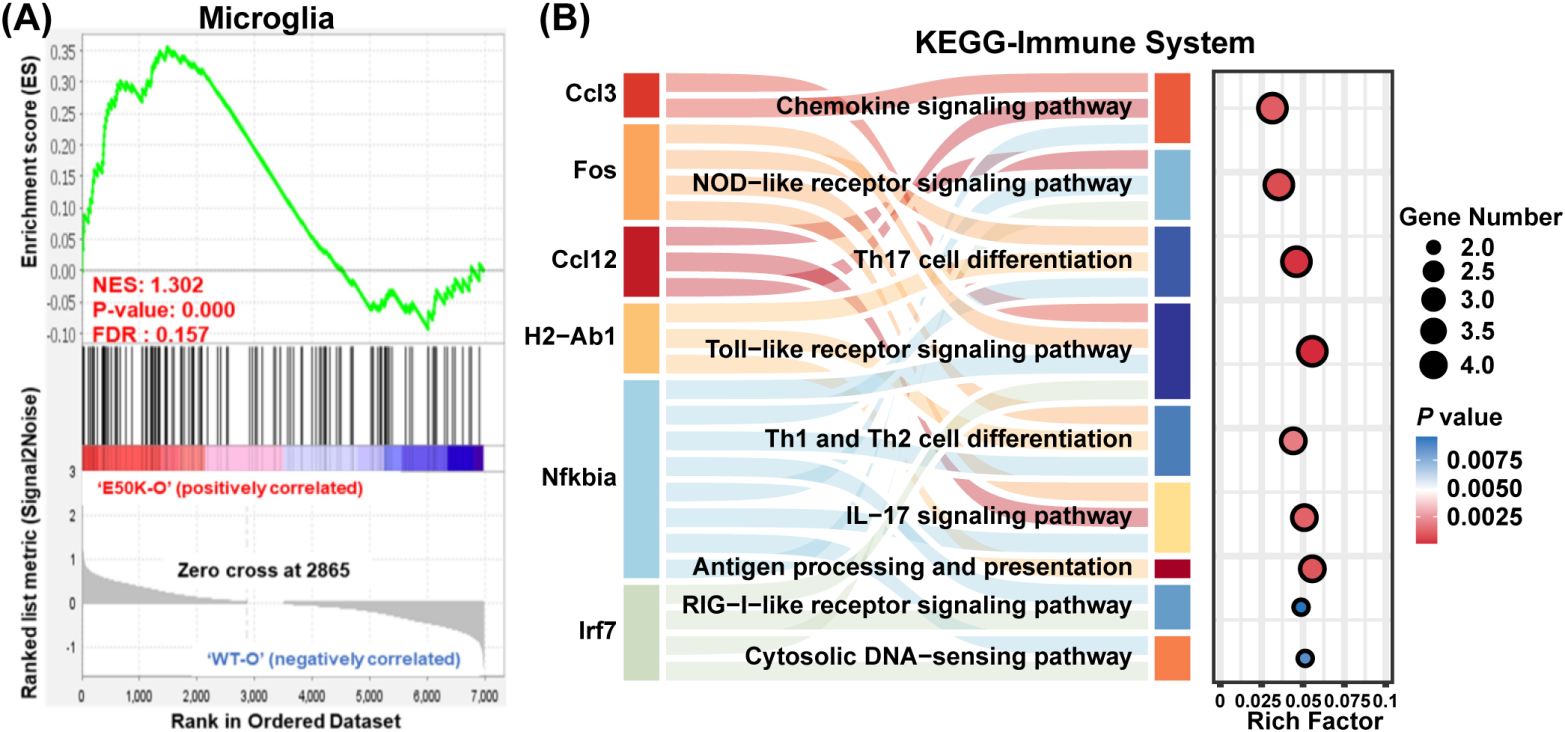
Analysis of gene enrichment and pathway involvement in microglia of E50K mutant mice. **(A)** The GSEA plot shows gene enrichment in the microglia of E50K mutant mice; **(B)** Functional enrichment analysis of differentially expressed genes in microglia of E50K mutant mice.

Proteomics analysis revealed that differentially expressed proteins in the retina of 18-month-old E50K mutant mice were significantly enriched in microglia (**Figure 3A**). To further validate the specific role of microglia in the mechanism of optic nerve damage in NTG, this study utilized scRNA-seq technology to analyze microglial populations in the retinas of E50K mutant and age-matched WT mice, focusing on their functional alterations.

Differential functional enrichment analysis of genes expressed in microglia from 18-month-old E50K mutant and WT mice highlighted significant enrichment in inflammatory-related signaling pathways (**Figure 3B**). The results showed that in the microglia of E50K mutant mice, several pathways, including the Chemokine Signaling Pathway, NOD-like Receptor Signaling Pathway, IL-17 Cell Differentiation, and Toll-like Receptor Signaling Pathway were notably enriched. These findings underscore the activation of microglia as a major driver of neuroinflammatory responses in the retinas of E50K mutant mice.

**Figure 4:**
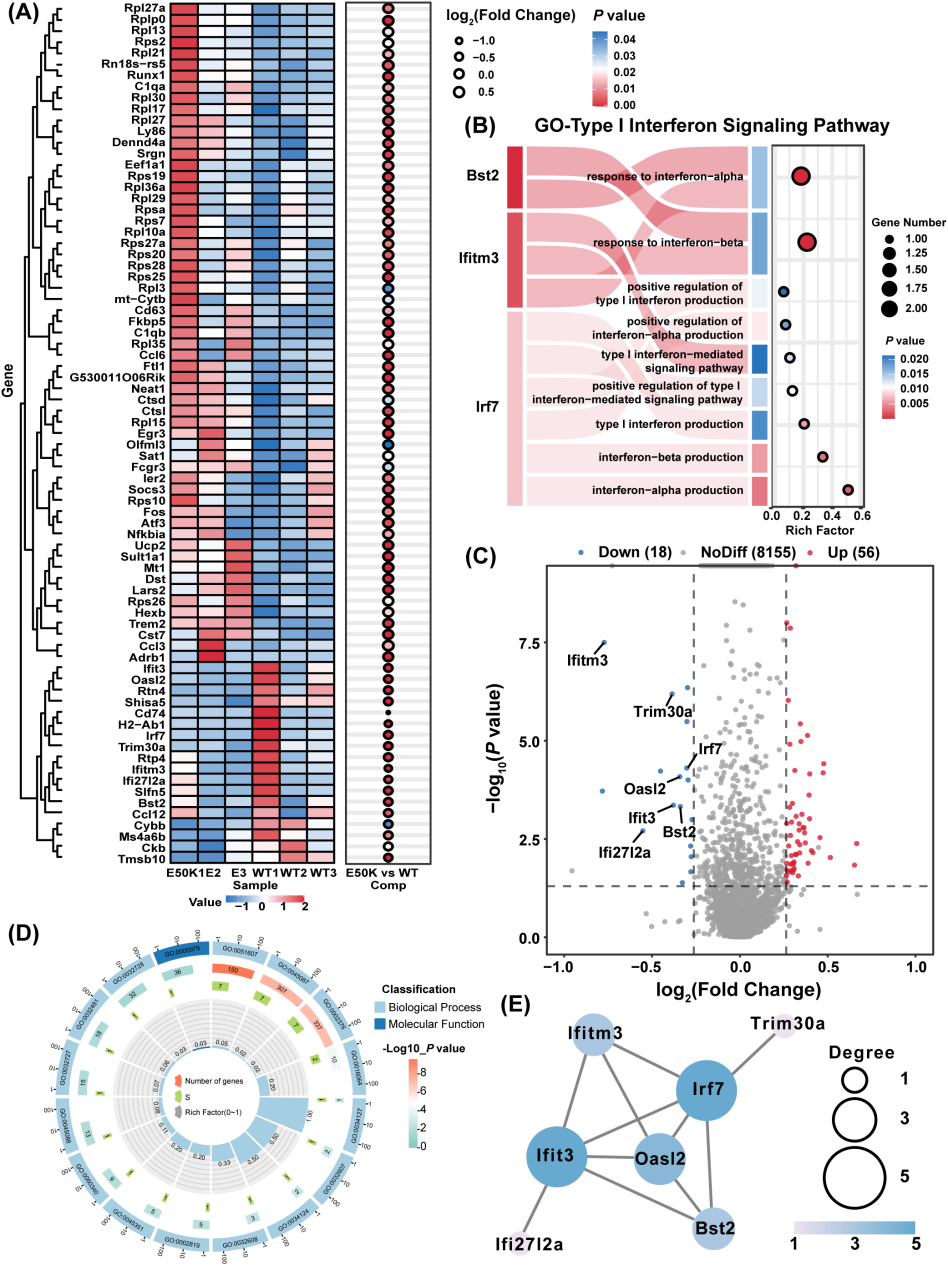
Differentially expressed genes (DEGs) and functional enrichment analysis in the retina of E50K mutant mice and the role of IRF7 in immune pathways and its interaction network. **(A)** Heatmap showing the expression of 74 DEGs in the retinas of E50K mutant and WT mice; **(B)** GO enrichment analysis highlighting the significant downregulation of the type I interferon signaling pathway; **(C)** Volcano plot displaying the distribution of DEGs in the E50K mutant group, with IRF7 showing notably reduced expression; **(D)** GO analysis indicates the role of IRF7 in immune pathways; **(E)** STRING database analysis reveals the interaction network of IRF7 in microglial cells.

Further analysis identified 74 differentially expressed genes (DEGs) in the retinas of 18-month-old E50K mutant and WT mice (**Figure 4A**). GO pathway enrichment analysis of these DEGs revealed a significant downregulation of the type I interferon signaling pathway in microglia from E50K mutant mice (**Figure 4B**). Several related genes were found to be downregulated, with the key regulatory factor IRF7 showing a particularly significant reduction (**Figure 4C**). IRF7 downregulation was closely associated with multiple inflammatory pathways (**Figure 3B**). As a central regulator of the type I interferon signaling pathway, the downregulation of IRF7 not only reduces the activity of this pathway but also exacerbates neuroinflammation in the retina through interactions with other inflammatory mediators. Functional enrichment analysis indicated that, in addition to IRF7 downregulation, multiple genes involved in the type I interferon signaling pathway underwent significant changes, with strong associations to inflammation, immune responses, and apoptosis.

IRF7 is involved in a variety of immune-related pathways, and GO analysis highlighted its extensive roles in immune and antiviral responses. Other significant pathways include viral defense responses, innate immune responses, and regulation related to MyD88-dependent and independent Toll-like receptor signaling pathways (**Figure 4D**). Furthermore, analysis using the STRING database confirmed the regulatory relevance of IRF7 in microglia and revealed a predicted interaction between IRF7 and Trim30a—a known negative regulator of NLRP3 expression (**Figure 4E**). Although our study did not further investigate Trim30a’s role, its concurrent downregulation with IRF7 hints at a potential link between IRF7 and NLRP3 activation. Based on this observation, we further explored whether IRF7 may be directly involved in modulating microglial inflammation via the NLRP3 signaling pathway.

### 3.4 Expression and functional analysis of IRF7 and NLRP3

**Figure 5:**
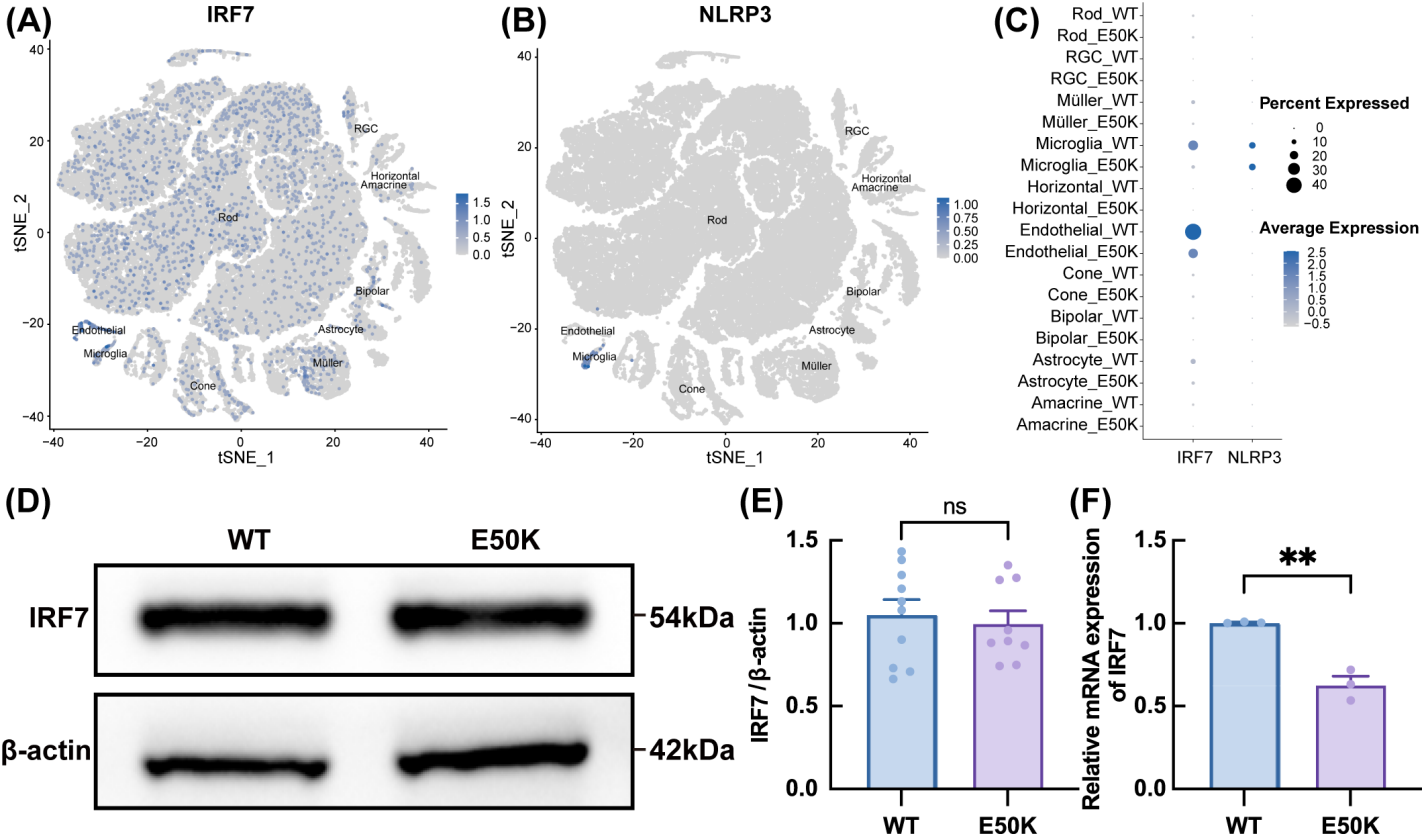
Expression and localization analysis of IRF7 and NLRP3 in retinal cells of E50K mutant and WT mice. **(A)** Expression distribution of IRF7 across different cell types; **(B)** Expression distribution of NLRP3 across different cell types; **(C)** Expression abundance of IRF7 and NLRP3 in various cell types; **(D, E)** Protein expression levels of IRF7 in the retinas of E50K mutant and WT mice; n≥9 **(F)** Relative mRNA expression levels of IRF7 in the retinas of E50K mutant and WT mice; n=3. **P < 0.01.

ScRNA-seq analysis revealed that NLRP3 is predominantly expressed in microglia within the retina, indicating its specific involvement in retinal immune response and inflammatory processes. In contrast, the expression of IRF7 is more widespread, being found not only in microglia but also in other retinal cell types (**Figures 5A–C**). To further investigate IRF7 expression at both the protein and transcript levels, retinal tissues from 18-month-old WT and E50K mutant mice were analyzed by Western blotting and quantitative PCR. As shown in **Figures 5D, E**, Western blot analysis revealed a slight reduction in IRF7 protein levels in the E50K group compared to WT, although the difference was not statistically significant. In contrast, qPCR results showed a significant decrease in IRF7 mRNA expression in E50K retinas (**Figure 5F**), indicating potential transcriptional downregulation of IRF7 in the mutant condition.

**Figure 6:**
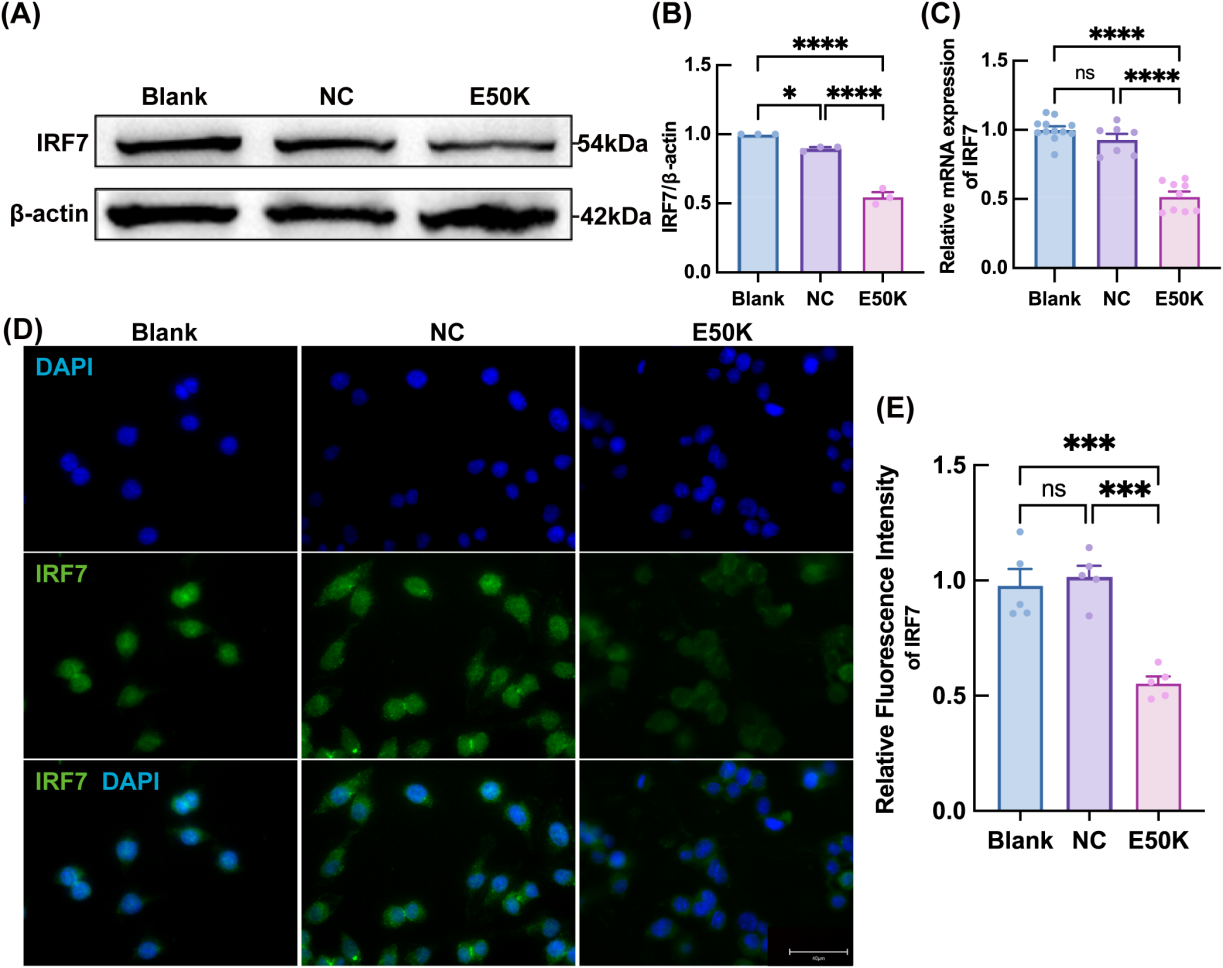
Verification of IRF7 expression in the E50K-BV2 cell line. **(A, B)** IRF7 protein expression levels in BV2 cells; n≥7 **(C)** Relative mRNA expression levels of IRF7 in BV2 cells; n=3 **(D, E)** IF staining of IRF7; n=5. *P < 0.05; ***P < 0.001; ****P < 0.0001.

We constructed an OPTN (E50K) mutant microglial cell line (E50K-BV2) for a more focused investigation of IRF7’s role in microglia. In the E50K mutant group, both IRF7 protein and mRNA expression levels were significantly reduced (**Figures 6A–C**), suggesting that the E50K mutation may impact IRF7 expression in microglia. Immunofluorescence (IF) staining further confirmed this finding, revealing a significant reduction in the fluorescence intensity of IRF7 in the E50K mutant group compared to the control group (**Figures 6D, E**), indicating suppressed expression of IRF7 in E50K mutant microglia.

**Figure 7:**
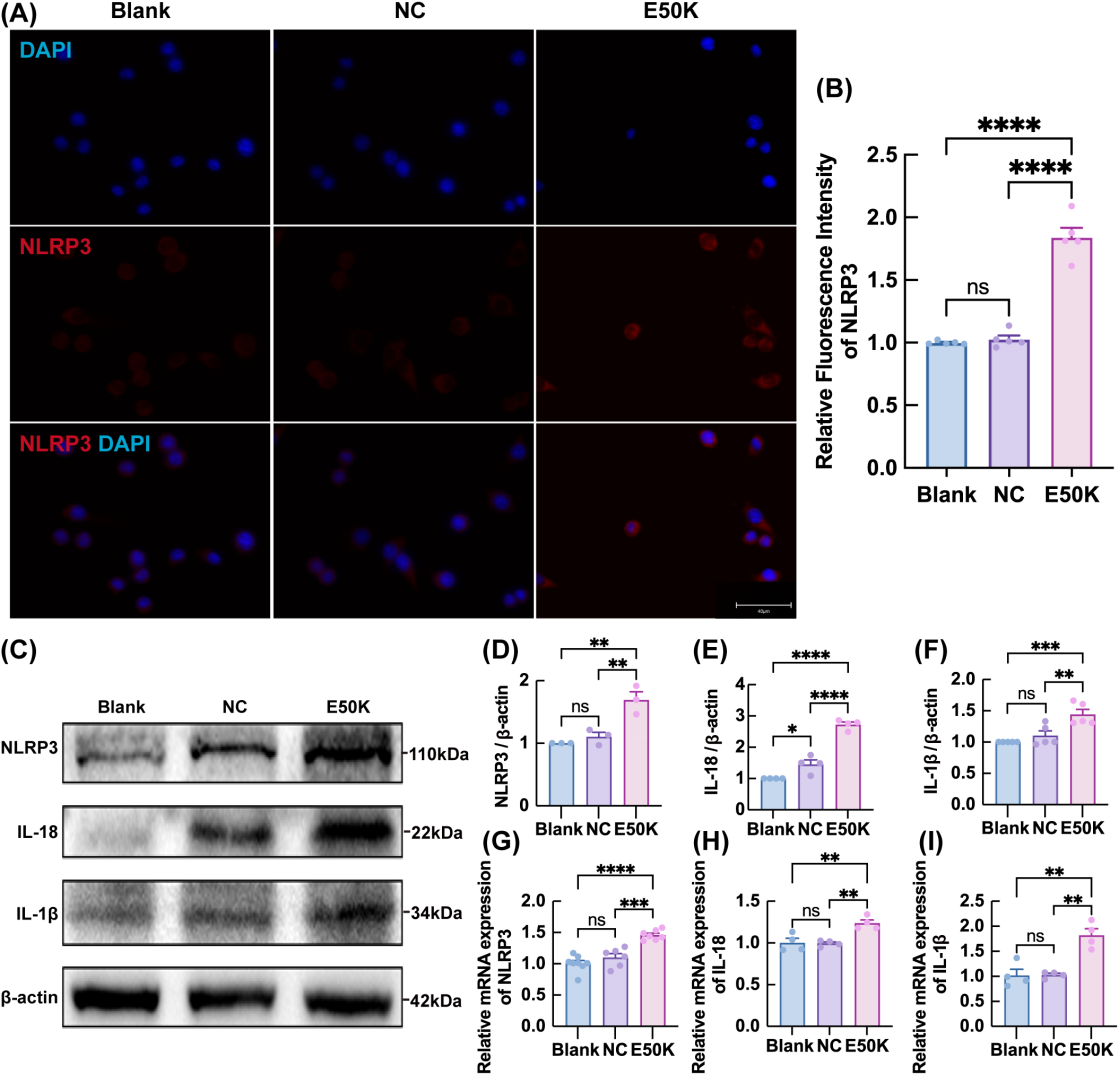
Effect of E50K mutation on the expression of NLRP3 and its downstream pro-inflammatory factors. **(A, B)** IF staining of NLRP3; n=5 **(C–I)** Protein and mRNA expression levels of NLRP3 and related pro-inflammatory factors in BV2 cells. WB: NLRP3: n = 3; IL-18: n = 4; IL-1β: n = 5; qPCR: NLRP3: n ≥ 6; IL-18: n ≥ 4; IL-1β: n ≥ 4. **P < 0.01; ***P < 0.001; ****P < 0.0001.

In contrast, NLRP3 expression was significantly enhanced in the E50K mutant group, with a marked increase in fluorescence intensity compared to the control group (**Figures 7A, B**). Consistent with our previous report in the same OPTN (E50K) NTG model, we again observed up-regulation of NLRP3 and its downstream cytokines IL-1β and IL-18 in E50K retinas. This suggests that the E50K mutation, which has previously been shown to induce microglial activation in the retina (5), may further facilitate this process through the upregulation of NLRP3. Furthermore, WB and qPCR analysis showed that the expression levels of NLRP3 and its associated pro-inflammatory factors, IL-18 and IL-1β, were significantly elevated in the E50K mutant group (**Figures 7C–I**).

### 3.5 Protein interaction analysis of IRF7 and NLRP3

**Figure 8:**
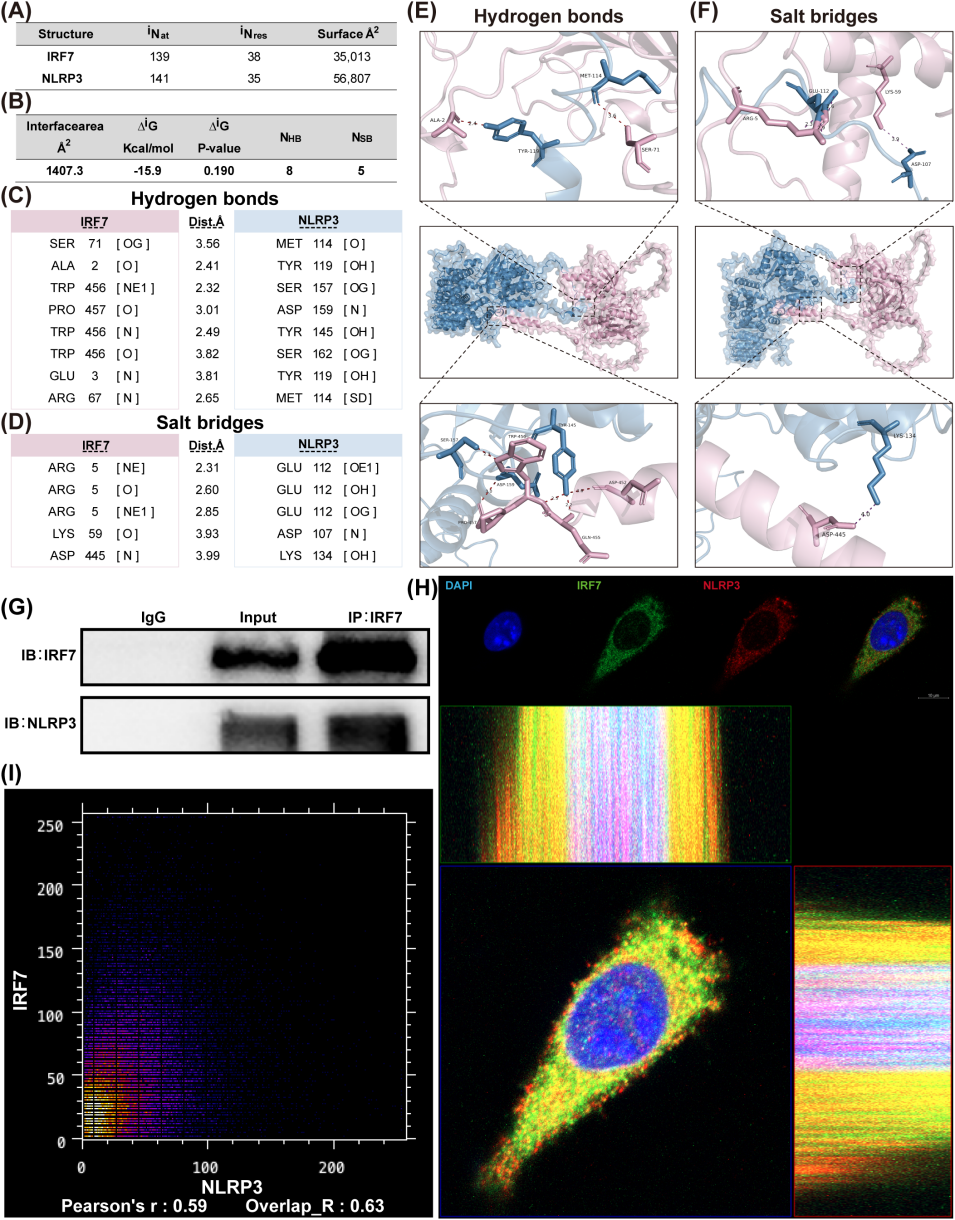
Molecular Docking and Interaction Analysis of IRF7 and NLRP3. **(A)** Structural data of IRF7 and NLRP3 proteins; **(B)** Thermodynamic parameters of the IRF7-NLRP3 binding interface; **(C-D)** Locations of hydrogen bond formation and salt bridge formation; **(E–F)** PyMOL visualization showing the 3D structure of IRF7 and NLRP3 and key interaction sites; **(G)** Co-immunoprecipitation (Co-IP) results showing the interaction of IRF7 and NLRP3 in E50K-BV2 cells; **(H)** IF colocalization shows the colocalization of IRF7 (green) and NLRP3 (red) in E50K-BV2 cells, with DAPI (blue) marking the cell nucleus. The merged images illustrate their co-localization in the cytoplasm; **(I)** The scatter plot shows a Pearson correlation coefficient (R) of 0.59 and an Overlap_R coefficient of 0.63, indicating moderate colocalization of IRF7 and NLRP3 in cells.

The interaction between IRF7 and NLRP3 was assessed through molecular docking analysis using publicly available protein data from the UniProt database. PDBePISA analysis revealed that the binding interface free energy change (Δ^i^G) between IRF7 and NLRP3 was -15.9 kcal/mol, suggesting that their binding is both spontaneous and stable (**Figure 8A**). Moreover, the total area of the binding interface was measured to be 1407.3Å², indicating a strong interaction between the two proteins (**Figure 8B**).

Further analysis revealed that IRF7 and NLRP3 formed eight hydrogen bonds (**Figure 8C**) and five salt bridges (**Figure 8D**), all of which enhanced the stability of their binding. Structural visualization using PyMOL confirmed the significant interaction between IRF7 and NLRP3, providing molecular evidence for their functional association in cellular inflammatory responses (**Figures 8E, F**).

To validate the interaction between IRF7 and NLRP3 in the E50K mutant group, an immunoprecipitation (Co-IP) experiment was performed. After capturing IRF7 with magnetic beads, WB analysis detected an NLRP3 band in the immunoprecipitation (IP: IRF7) group, indicating that IRF7 and NLRP3 form a complex in E50K-BV2 cells (**Figure 8G**).

IF staining of E50K-BV2 cells showed significant colocalization of IRF7 and NLRP3 in the cytoplasm of microglia. The overlapping areas of IRF7 (green) and NLRP3 (red) were visible in the cytoplasm, indicating a shared distribution of the two proteins within microglia (**Figure 8H**). Further image analysis revealed a Pearson’s correlation coefficient of 0.59 and an overlap coefficient of 0.63, suggesting a moderate degree of colocalization between IRF7 and NLRP3 in the cell (**Figure 8I**). This result supports the hypothesis that the two proteins interact within the same cellular compartment or participate in the same signaling pathway. Moreover, it suggests that the activation of the NLRP3-related inflammatory pathway may be closely linked to the downregulation of IRF7 expression.

## 4 Discussion

Glaucoma is the leading cause of irreversible blindness worldwide and is primarily characterized by RGCs apoptosis and axonal degeneration (11, 12). NTG is a subtype of glaucoma in which optic nerve damage occurs despite intraocular pressure remaining within the normal range. NTG is especially prevalent in Asian populations, accounting for over 60% of POAG cases. Unlike glaucoma with high intraocular pressure, intraocular pressure in NTG patients consistently stays within the normal range, rendering conventional intraocular pressure-lowering treatments less effective. While controlling intraocular pressure may slow the disease progression, neurodegeneration remains largely unavoidable. Therefore, understanding the molecular mechanisms of NTG-related optic nerve damage and identifying therapeutic targets are critical for improving clinical outcomes and guiding the development of novel treatment strategies.

Although the precise mechanisms underlying optic nerve damage in NTG remain incompletely understood, accumulating evidence suggests that both genetic factors and immune-mediated neuroinflammation contribute significantly to disease progression. Among these factors, the OPTN (E50K) mutation has been identified as a major pathogenic variant in NTG. OPTN is implicated in several neurodegenerative diseases and plays essential roles in cellular processes such as autophagy, inflammatory regulation, and maintenance of cellular homeostasis. The E50K mutation is thought to impair these functions, primarily by promoting aberrant activation of microglia. This activation leads to the excessive release of pro-inflammatory cytokines, thereby driving sustained neuroinflammation and exacerbating RGCs damage (13, 14). This mechanism underscores the central role of microglia in the pathogenesis and progression of NTG.

### 4.1 Establishment and phenotypic validation of the OPTN (E50K) NTG mouse model

We successfully generated an OPTN (E50K) mutant mouse model using CRISPR/Cas9 gene-editing technology. Phenotypic assessments confirmed that this model faithfully recapitulates key features of NTG. Throughout disease progression, the intraocular pressure remains within the normal range, and no obvious structural abnormalities are observed in the anterior segment. In aged mice, the retina displays hallmark features of glaucoma, including RGCs loss and impaired visual function (4). In contrast, no significant pathological changes are observed at earlier stages. Notably, this model has been internationally recognized and cataloged in the animal database of the renowned biomedical research institution, Jackson Laboratory, USA (Registration Number: C57BL/6J-Optn^em1Hyua^ MGI: 7278809). This achievement provides a valuable biological tool for studying the mechanisms of NTG-induced optic nerve damage and potential therapeutic strategies, laying a solid foundation for further exploration of NTG’s pathological processes.

### 4.2 Single-cell transcriptomic profiling reveals retinal cell-type composition in aged E50K mutant mice

As research into NTG deepens, the complex roles of retinal cells in disease progression have become increasingly emphasized. In particular, dynamic changes and interactions among different retinal cell subpopulations in aged E50K mutant mice may play a key role in retinal neurodegeneration and visual function loss.

Traditional transcriptomic approaches based on bulk retinal tissue lack the resolution to distinguish individual cell types, limiting the detection of cell-specific molecular alterations. This challenge is particularly evident in animal models, where isolating distinct retinal subpopulations remains technically difficult. To address this, we employed scRNA-seq to generate high-resolution transcriptional profiles and investigate retinal cellular heterogeneity at single-cell resolution. Since retinal cells exhibit heterogeneous responses under pathological conditions, scRNA-seq offers a powerful approach to elucidate their distinct contributions to NTG-related optic nerve damage.

Recent studies, such as those by Voigt (15) et al. and Menon (16) et al., have demonstrated the power of scRNA-seq in identifying stress-responsive and disease-associated retinal cell types in models of glaucoma and optic nerve damage. These studies have revealed distinct microglial activation profiles and RGC subtypes susceptible to neurodegeneration. However, most prior research has focused on acute or high intraocular pressure models. In contrast, our study provides the first single-cell transcriptomic insight into an NTG model carrying the OPTN (E50K) mutation, providing a more accurate reflection of the disease’s clinical features.

In this study, we profiled retinal tissues from aged E50K mutant and WT mice using scRNA-seq technology, successfully isolating multiple cell subpopulations. By integrating these subpopulations based on common marker genes, we identified ten distinct cell types (including Amacrine, Astrocyte, Bipolar, Cone, Endothelial, Horizontal, Microglia, Müller, RGCs, and Rod). For the first time, a retinal cell subpopulation map of aged E50K mutant mice was created by our group, which is crucial for accurately understanding the specific roles of retinal subpopulations in optic nerve injury in elderly E50K mutant mice.

### 4.3 Microglia-specific enrichment of differential proteins in the retina of aged E50K mutant mice

However, gene expression profiles alone are insufficient to fully elucidate the molecular mechanisms underlying pathological changes. As the final effectors of cellular processes, proteins dynamically reflect functional and pathological alterations through changes in expression and post-translational regulation. Therefore, to further investigate how the E50K mutation impacts the function of specific retinal cell subpopulations in aged mice, this study focused on differential proteins in the retina. Through protein-level analysis, we aimed to reveal key molecular pathways and potential disease-driving factors.

Our proteomic analysis revealed that, compared to WT mice, differentially expressed proteins in the retinas of E50K mutant mice were significantly enriched in microglia, with this enrichment being especially pronounced in aged mice. In addition, in the retinal ganglion cell layer of aged E50K mutant mice, the number of Iba-1^+^ microglia was significantly increased (5). These findings suggest that the OPTN (E50K) mutation leads to microglial activation. The timing of these results aligns with the appearance of NTG optic nerve damage phenotypes in E50K mutant mice.

Recent proteomics-based studies have highlighted early microglial involvement in glaucomatous neurodegeneration. For example, Lin reported an upregulation of microglia-associated proteins, such as Iba1 and CD11b, in the retina and optic nerve head of rats subjected to ocular hypertension, suggesting early microglial activation prior to detectable neuronal damage (17). Our research utilizes 18-month-old OPTN (E50K) mutant mice with normal intraocular pressure, enabling the investigation of protein-level alterations and microglial activation during the progression of NTG.

Microglia are the innate immune cells of the retina, responsible for surveilling the neural environment and maintaining tissue homeostasis (18, 19). However, previous studies have shown that during the pathological progression of high intraocular pressure glaucoma, microglia often become excessively activated and play a pivotal role in both neuroinflammation and neuronal damage (20). In particular, at the early stages of the disease, this activation leads to the excessive release of pro-inflammatory cytokines, which not only exacerbate RGC apoptosis but also further accelerate optic nerve degeneration (21, 22).

### 4.4 Inflammatory activation in E50K mutant microglia is associated with IRF7 downregulation

Functional enrichment analysis further revealed that inflammation-related pathways were significantly enriched in microglia from aged E50K mutant mice. Our previous work suggests that the OPTN (E50K) mutation may trigger intracellular inflammatory cascades through NLRP3 activation. This activation can convert microglia to a pro-inflammatory phenotype, resulting in the release of large amounts of pro-inflammatory cytokines, which contribute to a neuroinflammatory microenvironment and exacerbate RGCs damage and apoptosis (9). NLRP3 inflammasome activation contributes directly to retinal ganglion cell death and is considered a crucial target in glaucomatous neurodegeneration (23, 24).

Consistent with our findings, previous studies have shown that microglia can aggravate retinal inflammation and RGCs damage via activation of the NLRP3 inflammasome in glaucoma models (25, 26). These results reinforce the idea that microglia contribute to neuroinflammatory responses by activating the NLRP3 inflammasome, which ultimately leads to optic nerve damage (27). Therefore, building on this foundation, this study investigates how the OPTN (E50K) mutation exacerbates NTG optic nerve damage through pro-inflammatory activation mechanisms in microglia.

ScRNA-seq and bioinformatics analysis revealed a significant enrichment of inflammatory pathways in the OPTN (E50K) mutant group, indicating enhanced inflammatory activity at the cellular level. Furthermore, the OPTN (E50K) mutation may contribute to the inflammatory response induced by microglia by inhibiting the type I interferon pathway, thereby indirectly exacerbating optic nerve damage. Functional enrichment analysis showed significant downregulation of regulatory factors in the type I interferon signaling pathway, particularly the key regulator IRF7. This suggests that IRF7 plays an essential role in modulating the inflammatory response in microglia. IRF family members such as IRF7 and IRF8 are known to shape microglial identity and epigenetic state, and their disruption shifts the transcriptome toward a disease-associated phenotype (28).

Type I interferon is a key regulator that activates downstream signaling pathways and amplifies the inflammatory response (29). Within this pathway, IRF7 serves as a major transcription factor that plays a critical role in suppressing pro-inflammatory cytokine expression while enhancing neuroprotective mechanisms. In addition to its role in the type I interferon pathway, IRF7 is involved in multiple immune-related signaling cascades, highlighting its broad regulatory capacity in inflammatory and immune responses. Furthermore, analysis using the STRING database revealed a predicted interaction between IRF7 and Trim30a, a gene that is also downregulated in our model and has been shown to negatively regulate NLRP3 expression (30). This supports a model in which both IRF7 and Trim30a deficiency synergistically promote NLRP3 overactivation, potentially amplifying the inflammatory cascade in microglia. These findings suggest that IRF7 may influence inflammation through canonical interferon signaling. This potentially amplifies inflammatory responses in the retina and contributes to the damage of RGCs during NTG progression. Previous studies have indicated that the downregulation of IRF7 is associated with reduced NLRP3 expression (31), but these observations were primarily based on pathway-level regulatory analyses. Research by Cohen and Deng (32, 33) demonstrated that under inflammatory conditions, IRF7 expression is negatively correlated with the activation of the NF-κB pathway, which subsequently promotes NLRP3 expression (34). Moreover, studies by Lu (35) found that silencing MEF2D, a transcription factor involved in gene expression regulation, leads to increased NLRP3 mRNA expression and decreased IRF7 mRNA expression, suggesting a regulatory axis connecting these molecules. Together, these studies point to an indirect relationship between IRF7 and NLRP3. However, whether a direct molecular interaction exists between IRF7 and NLRP3 remains unclear and warrants further investigation.

In the animal model, although IRF7 mRNA was significantly reduced in aged E50K retinas, Western blot showed no corresponding decrease in protein levels. This discrepancy likely reflects the cellular heterogeneity of the retina, where IRF7 is expressed across multiple cell types. As microglia constitute only a small fraction of retinal cells, their specific downregulation of IRF7 may be masked at the tissue level. Moreover, post-transcriptional regulation may cause a lag or dissociation between transcript and protein expression. In contrast, qPCR is more sensitive to transcript-level changes and can detect subtle differences that may not be reflected in protein abundance. To verify whether the observed changes originate specifically from microglia, we employed the BV2 microglial cell line and found that both IRF7 mRNA and protein levels were significantly downregulated in E50K-mutant cells. These findings provide direct, cell-specific evidence that the suppression of IRF7 is attributable to microglial alterations and underscore the limitations of bulk tissue analysis in detecting such cell-type-specific molecular changes.

Several upstream mechanisms may help explain why the OPTN (E50K) mutation leads to reduced IRF7 expression in microglia. The E50K variant is known to impair autophagy and mitophagy, resulting in the accumulation of dysfunctional mitochondria and elevated cellular stress (36, 37). Such mitochondrial and autophagic defects generate chronic oxidative and inflammatory stress—conditions reported to repress interferon-dependent transcriptional regulators, including members of the IRF family (38, 39).

Collectively, these findings support a plausible mechanism in which E50K-induced mitochondrial dysfunction and impaired autophagy converge to suppress IRF7 expression in microglia, thereby lowering the threshold for subsequent NLRP3 inflammasome activation.

Given the established role of NLRP3 in mediating microglial inflammatory responses during NTG-related optic nerve damage, we further investigated whether IRF7 may directly interact with NLRP3 and modulate its activation in microglia.

Extensive evidence links IRF7-driven type I interferon signaling to NLRP3 inflammasome biology. IRF7, the key transcription factor for IFN-I responses (39), and IFN-I has been shown to restrain NLRP3 priming and IL-1β maturation through multiple inhibitory mechanisms (40). Reduced IRF7 expression may therefore diminish these IFN-I–dependent suppressive effects on NLRP3 induction. In parallel, IFN-I signaling helps maintain mitochondrial homeostasis and limits mitochondrial ROS generation (41). Together, these findings suggest that IRF7 downregulation may create a cellular environment more permissive to NLRP3 activation.

Beyond these indirect mechanisms, our data reveal a direct cytoplasmic interaction between IRF7 and NLRP3. This is notable because inflammasome activation critically depends on protein–protein interactions: regulators such as NEK7 (42) or TXNIP (43) modulate NLRP3 oligomerization, conformational readiness, and subcellular organization through direct binding. By analogy, the IRF7–NLRP3 interaction identified here raises the possibility that IRF7 may influence NLRP3 assembly dynamics or spatial coordination within microglia, adding a mechanistic layer distinct from transcriptional regulation.

Together, these findings support a plausible mechanistic hypothesis in which IRF7 downregulation not only facilitates NLRP3 priming, but more importantly, may influence NLRP3 activation through its direct interaction with NLRP3. This interaction may affect the interaction interface and assembly dynamics required for inflammasome formation, thereby amplifying inflammatory signaling in E50K microglia.

To further substantiate the relationship between IRF7 dysregulation and NLRP3-associated inflammatory activation, we systematically evaluated inflammasome-related pathways using our single-cell dataset and supplemental experiments (**Supplementary Figure S1**). Although individual inflammasome genes (NLRP3, PYCARD, CASP1) exhibited only mild upward trends in E50K microglia, likely due to the limited sample size (n = 3 per group) and intrinsic dropout in scRNA-seq (**Supplementary Figure S1B**), the overall directionality indicated a shift toward a more pro-inflammatory state. The upstream transcription factor IRF7 was markedly reduced in E50K microglia (**Supplementary Figure S1A**), consistent with our previous bulk and *in-vivo* observations showing suppression of type I IFN signaling in this model.

Because individual inflammasome genes are lowly expressed in single-cell datasets, we next performed pathway-level analyses using a NLRP3-inflammasome gene set (NLRP3, PYCARD, CASP1, IL-1β, IL-18, GSDMD). GSVA/module scoring revealed a consistent, though non-significant, elevation in inflammasome activity in E50K microglia (**Supplementary Figure S1C**), supporting the presence of low-grade priming rather than overt activation at the single-cell level.

Importantly, GSEA revealed significant enrichment of two canonical inflammasome-priming pathways: Toll-like receptor signaling pathway (NES = 2.00, FDR = 0.002) and TNF signaling pathway (NES = 1.91, FDR = 0.006) in E50K microglia (**Supplementary Figures S1D, E**). These pathways represent the essential “signal 1” inputs that drive NF-κB–dependent induction of NLRP3 and pro-IL-1β, suggesting that the E50K microglial environment is transcriptionally primed for enhanced inflammasome readiness. Consistent with these transcriptomic findings, TNF-α protein levels were significantly increased in E50K-BV2 cells (**Supplementary Figures S1F, G**), providing biochemical confirmation of upstream inflammatory activation. Taken together, these supplemental results indicate that although single-cell expression changes of individual inflammasome genes remain modest, E50K microglia exhibit a robustly primed inflammatory state, characterized by suppression of IFN-I/IRF7 signaling and concomitant activation of NF-κB–associated pathways that collectively lower the threshold for NLRP3 inflammasome activation.

### 4.5 IRF7 interacts with NLRP3 and regulates inflammatory responses in E50K mutant microglia

To clarify how IRF7 and NLRP3 contribute to the inflammatory response induced by the OPTN (E50K) mutation in microglia, we integrated evidence from scRNA-seq analysis, *in vitro* experiments, and bioinformatics approaches.

First, scRNA-seq analysis of aged E50K mutant mice revealed alterations in the expression levels of IRF7 and NLRP3 within microglial clusters. Functional enrichment analysis further suggested that these changes may be associated with inflammatory signaling pathways, including the type I interferon axis. These findings are consistent with our previous study (9), which demonstrated that the OPTN (E50K) mutation activates the NLRP3 inflammasome, leading to a cascade of retinal inflammation and structural damage, and that NLRP3 inhibition via MCC950 effectively mitigates this process and improves visual function. The present study builds upon these findings by exploring the upstream regulatory mechanisms of NLRP3 activation in microglia, particularly focusing on IRF7 as a potential suppressor.

Moreover, the upregulation of NLRP3 has been widely reported in activated microglia in various neurodegenerative diseases, such as Alzheimer’s disease (AD) and multiple sclerosis (MS) (44), suggesting a shared inflammatory mechanism. Ising (45) demonstrated that activation of the NLRP3 inflammasome in microglia accelerates tau pathology and cognitive impairment in an AD mouse model, providing direct evidence that innate immune activation contributes to neurodegeneration through inflammasome signaling. Similarly, Lee (46) highlighted the role of NLRP3 as a central driver of chronic neuroinflammation in both AD and Parkinson’s disease and suggested that natural product-derived NLRP3 inhibitors may offer therapeutic benefits. Zhang (47) further emphasized that excessive and persistent activation of NLRP3 leads to pyroptotic cell death and IL-1β/IL-18 release in the AD brain, reinforcing its pathological significance.

These findings are consistent with recent reports showing that NLRP3 inflammasome activation in microglia not only contributes to neuronal injury but also serves as a final common pathway across diverse neurodegenerative contexts, including glaucoma (48, 49).

These observations from central nervous system disorders closely parallel our findings in NTG. Based on these findings, we hypothesized that IRF7 and NLRP3 may interact or participate in a shared regulatory mechanism during the inflammatory response in NTG. In the E50K-mutant BV2 cells, we validated that both IRF7 protein and mRNA expression levels were significantly decreased, accompanied by increased expression of NLRP3 and its downstream inflammatory mediators IL-18 and IL-1β. These results provide direct evidence that the E50K mutation in microglia triggers a specific transcriptional and inflammatory cascade, supporting that IRF7 and NLRP3 play key roles in mediating the inflammatory phenotype associated with NTG. Interestingly, similar transcriptional suppression of interferon regulators like IRF7 has been observed in other optic neuropathies, which correlates with increased inflammasome activation and neuronal loss (50).

Next, to explore the molecular basis of this interaction, we performed protein-protein interaction (PPI) analysis using the STRING database, which predicted potential interactions between IRF7 and known NLRP3 regulators, such as Trim30a, which has been shown to suppress NLRP3 activation in peripheral macrophages.

Furthermore, molecular docking revealed a potentially stable binding interface between IRF7 and NLRP3, characterized by a relatively large interaction surface area typically indicative of multiple contact points such as hydrogen bonds, hydrophobic interactions, and electrostatic forces. These interactions collectively contribute to the stability of the complex and imply a direct or indirect interaction that may influence inflammasome activation. Combined with these results, these findings support the hypothesis that IRF7 and NLRP3 are in close proximity within the cell and may act synergistically in the inflammatory signaling pathway. This interaction provides new experimental evidence that IRF7 may regulate NLRP3 activity in microglia. This view is consistent with the framework proposed by Scheiblich, who emphasized that dynamic cross-talk between innate immune pathways and glial cell activation plays a central role in age-related neuroinflammation and neurodegeneration, particularly in retinal tissues (51). Finally, we employed experimental validation to confirm this interaction. Confocal immunofluorescence and co-immunoprecipitation (Co-IP) demonstrated colocalization and potential binding between IRF7 and NLRP3 in the cytoplasm of E50K-mutant BV2 microglial cells, supporting the hypothesis that IRF7 may functionally regulate NLRP3 activation. These findings further substantiate the existence of a direct molecular interaction between the two proteins and reinforce their potential involvement in the inflammatory signaling pathway.

### 4.6 Summary and perspectives

In summary, our integrated findings from single-cell transcriptomic, proteomic, and functional analyses converge to outline a mechanistic model in which the OPTN (E50K) mutation downregulates IRF7 expression in microglia, thereby suppressing the type I interferon signaling pathway and leading to the activation of NLRP3 and its downstream inflammatory mediators. Inhibiting NLRP3 has been shown to attenuate retinal inflammation and protect against optic nerve injury in NTG models, reinforcing the importance of targeting this pathway before irreversible damage occurs (52). Furthermore, loss of the negative regulator A20 in microglia leads to excessive NLRP3 activation and central nervous system inflammation, emphasizing the importance of upstream regulation (53). These findings highlight the pathological relevance of NLRP3 activation and raise important questions about how upstream regulators like IRF7 influence this process. This process underscores the central role of both IRF7 and NLRP3 in regulating microglial inflammatory states and suggests a potential direct interaction between these two molecules, providing a valuable direction for future intervention target investigations.

Moreover, leveraging scRNA-seq, our study reveals the functional heterogeneity and complexity of microglia in NTG-related optic nerve damage. This observation is consistent with recent scRNA-seq analyses that uncover spatial and disease-associated microglial subtypes with distinct inflammatory signatures in neurodegenerative conditions, including Alzheimer’s and retinal diseases (54, 55). These insights not only enhance our understanding of NTG pathogenesis at the cellular and molecular levels but also lay the groundwork for developing precision therapeutic strategies that target microglial inflammation. Nevertheless, further *in vivo* validation using conditional knockout models is warranted to clarify the functional significance of the IRF7-NLRP3 interaction in NTG.

While our study provides novel insights into the IRF7-NLRP3 axis in microglial inflammation in NTG, several limitations should be acknowledged. First, although our molecular docking and Co-IP assays suggest a potential interaction between IRF7 and NLRP3, these findings are primarily based on *in vitro* and bioinformatic analyses. The direct regulatory relationship and mechanistic causality *in vivo* remain to be fully established. Second, the use of BV2 microglial cells and aged E50K mutant mice provides important mechanistic clues, but these models may not fully capture the complexity of NTG pathophysiology. Future studies employing microglia-specific conditional knockout models of IRF7 or NLRP3—both in cellular systems and animal models—will be essential to validate their functional relevance in retinal neuroinflammation and optic nerve damage. Moreover, the upstream regulatory mechanisms leading to IRF7 suppression in the context of NTG remain poorly understood and warrant further investigation. Despite these limitations, our findings lay a solid foundation for future *in vivo* validation and therapeutic exploration targeting the IRF7-NLRP3 signaling axis in NTG.

Together, these multi-dimensional findings-from transcriptomic profiling to *in vitro* validation and protein-protein interaction studies—support the notion that the E50K mutation promotes a shift toward a pro-inflammatory microglial phenotype by disrupting IRF7-NLRP3 signaling. This cascade ultimately exacerbates retinal neuroinflammation in NTG. The identification of the IRF7-NLRP3 axis as a potential regulatory node provides a promising target for future therapeutic interventions in NTG and other neuroinflammatory diseases.

## 5 Conclusion

In this study, we demonstrated that the OPTN (E50K) mutation promotes a pro-inflammatory microglial phenotype by downregulating IRF7 and activating the NLRP3 inflammasome, thereby contributing to optic nerve damage in NTG. Transcript-level and protein-level analyses confirmed reduced IRF7 expression in E50K-mutant microglia, accompanied by increased NLRP3 and downstream cytokines IL-1β and IL-18. Protein interaction and imaging studies further supported a direct interaction between IRF7 and NLRP3, implicating a disrupted IRF7-NLRP3 regulatory axis in disease progression. These findings suggest a novel microglial mechanism through which the E50K mutation may promote sustained retinal inflammation and neurodegeneration. Future research should explore upstream regulators of IRF7 and evaluate whether targeting this axis can attenuate retinal inflammation and preserve optic nerve function.

## Data Availability

The data presented in the study are deposited in the OMIX (Open Archive for Miscellaneous Omics Data) repository, accession number OMIX014170.

## Supplementary material

Supplementary Figure 1 Single-cell and biochemical evidence supporting activation of NLRP3-related inflammatory pathways in OPTN (E50K) microglia. **(A)** UMAP feature map showing reduced IRF7 expression in the retinal microglia of OPTN (E50K) mice compared to WT mice; **(B)** Vlnplots of IRF7, NLRP3, PYCARD (ASC) and CASP1 expression in microglia. Each dot represents a biological replicate (n=3 per group). Mild upward trends of NLRP3, PYCARD and CASP1 were observed in E50K microglia, although differences did not reach statistical significance; **(C)** GSVA enrichment scores for a NLRP3-inflammasome core gene set, showing a higher pathway score in E50K microglia but with no statistically significant difference; **(D, E)** GSEA plots demonstrating significant enrichment of Toll-like receptor signaling (NES = 2.00, FDR = 0.002) and TNF signaling (NES = 1.91, FDR = 0.006) pathways in E50K microglia, indicating enhanced inflammasome-priming activity; (F-G) TNF-α protein expression levels in BV2 cells. Data are mean ± SEM. **P* < 0.05, ****P* < 0.001 (one-way ANOVA with *post-hoc* test).
