# Supplementary figures and images for: Revealing the role of regulatory microglial IRF7-NLRP3 interactions in optic nerve damage of normal-tension glaucoma based on single-cell RNA sequencing
Source: Front Immunol. 2026 Jan 12;16:1700998. doi: 10.3389/fimmu.2025.1700998 (PMC12832388; doi:10.3389/fimmu.2025.1700998)

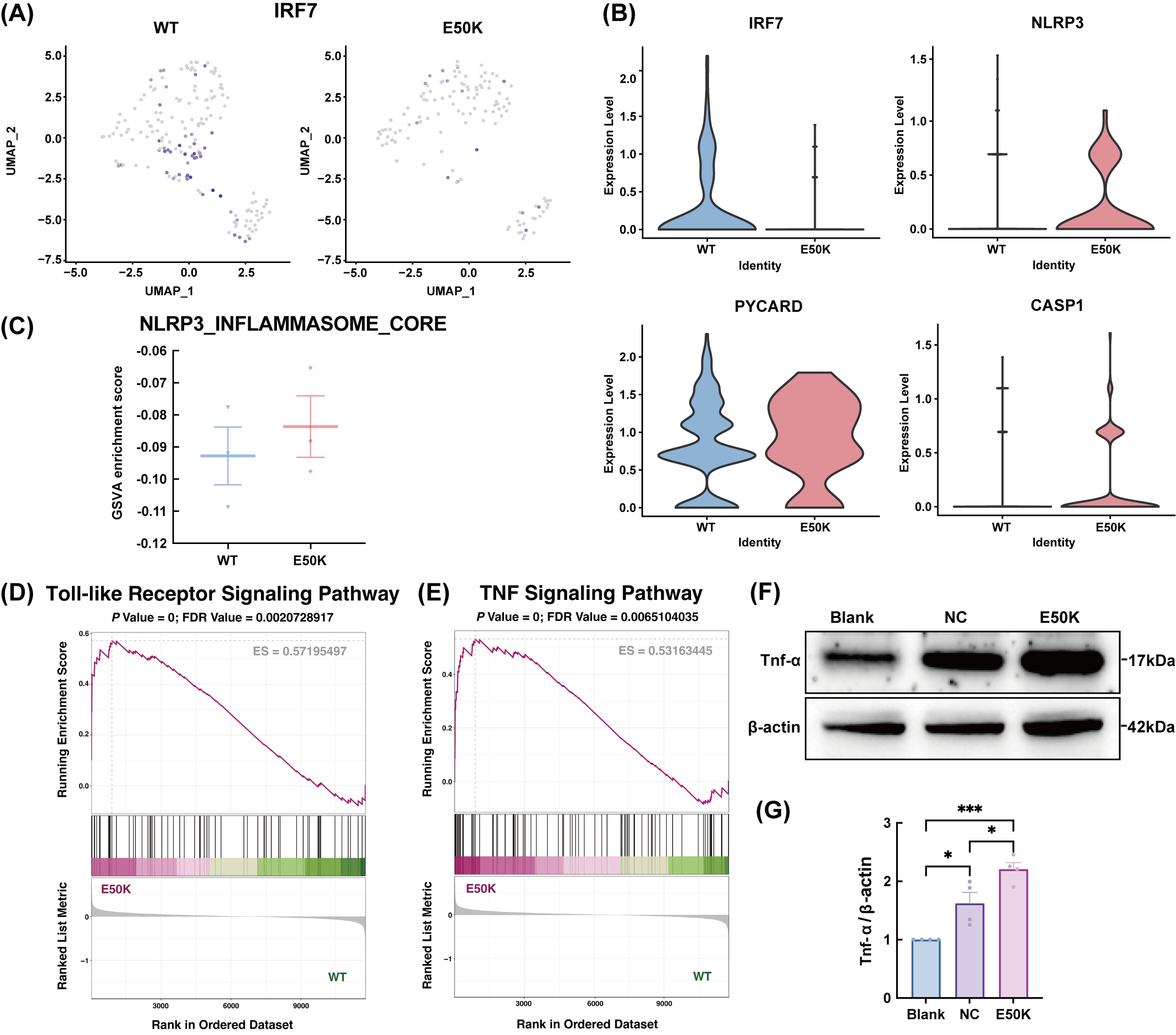

Supplement: Supplementary Figure 1 — Single-cell and biochemical evidence supporting activation of NLRP3-related inflammatory pathways in OPTN (E50K) microglia. (A) UMAP feature map showing reduced IRF7 expression in the retinal microglia of OPTN (E50K) mice compared to WT mice; (B) Vlnplots of IRF7, NLRP3, PYCARD (ASC) and CASP1 expression in microglia. Each dot represents a biological replicate (n=3 per group). Mild upward trends of NLRP3, PYCARD and CASP1 were observed in E50K microglia, although differences did not reach statistical significance; (C) GSVA enrichment scores for a NLRP3-inflammasome core gene set, showing a higher pathway score in E50K microglia but with no statistically significant difference; (D, E) GSEA plots demonstrating significant enrichment of Toll-like receptor signaling (NES = 2.00, FDR = 0.002) and TNF signaling (NES = 1.91, FDR = 0.006) pathways in E50K microglia, indicating enhanced inflammasome-priming activity; (F-G) TNF-α protein expression levels in BV2 cells. Data are mean ± SEM. *P < 0.05, ***P < 0.001 (one-way ANOVA with post-hoc test). [file Image1.tif]

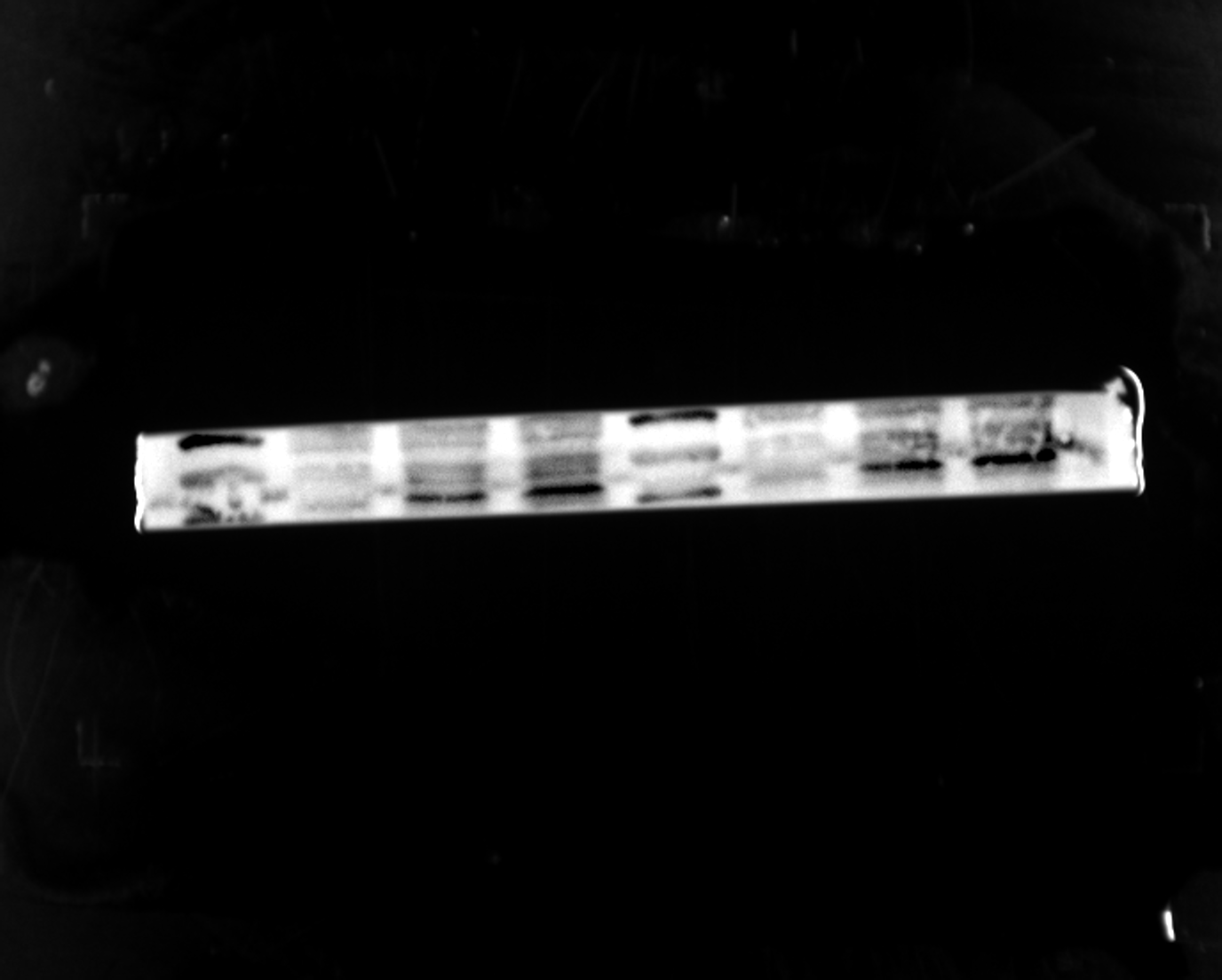

Supplement: Supplementary file 2 [file DataSheet1.zip › Supplementary file/Figure7c-IL-18-merge.Tif]

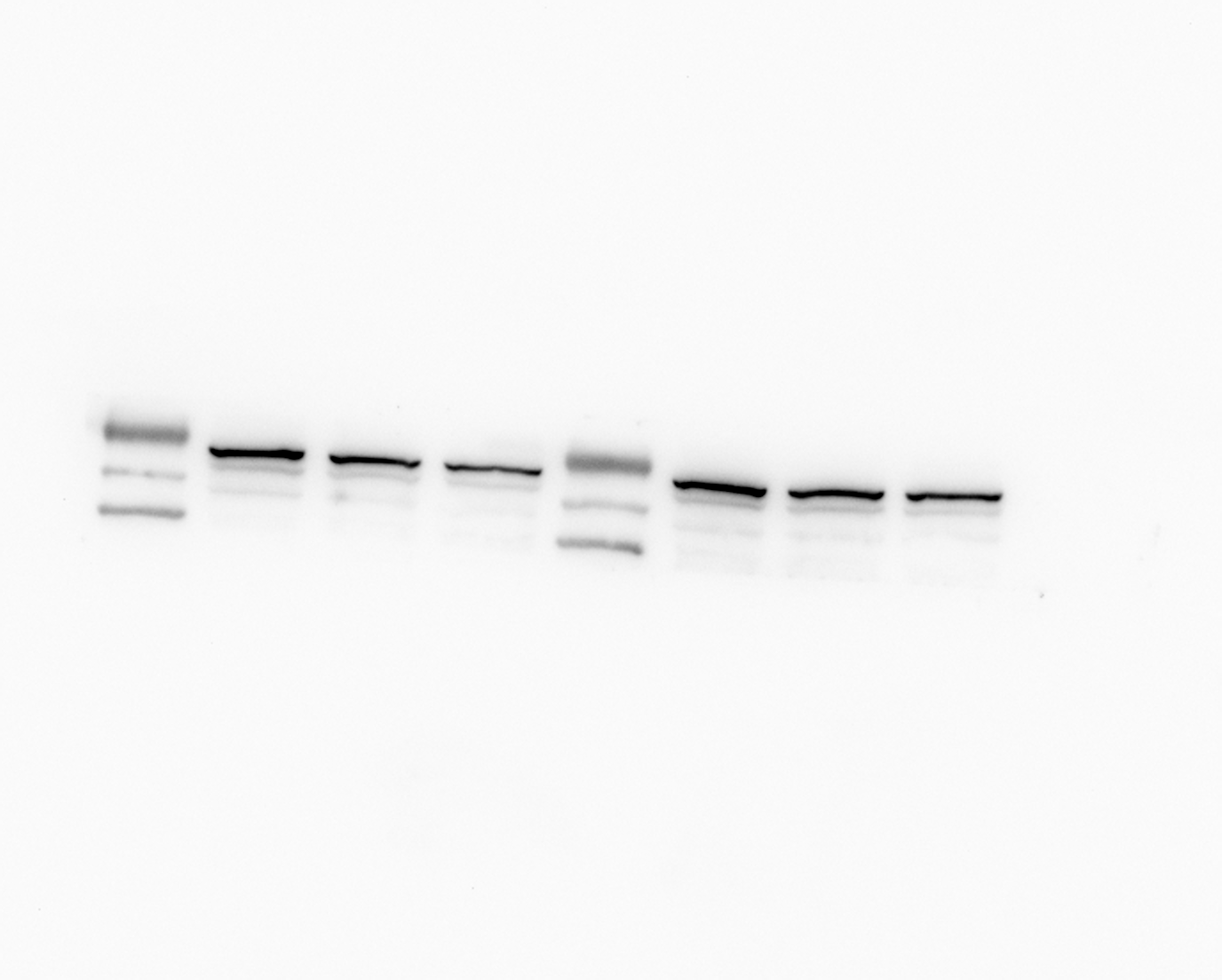

Supplement: Supplementary file 2 [file DataSheet1.zip › Supplementary file/Figure6a-IRF7-1.tif]

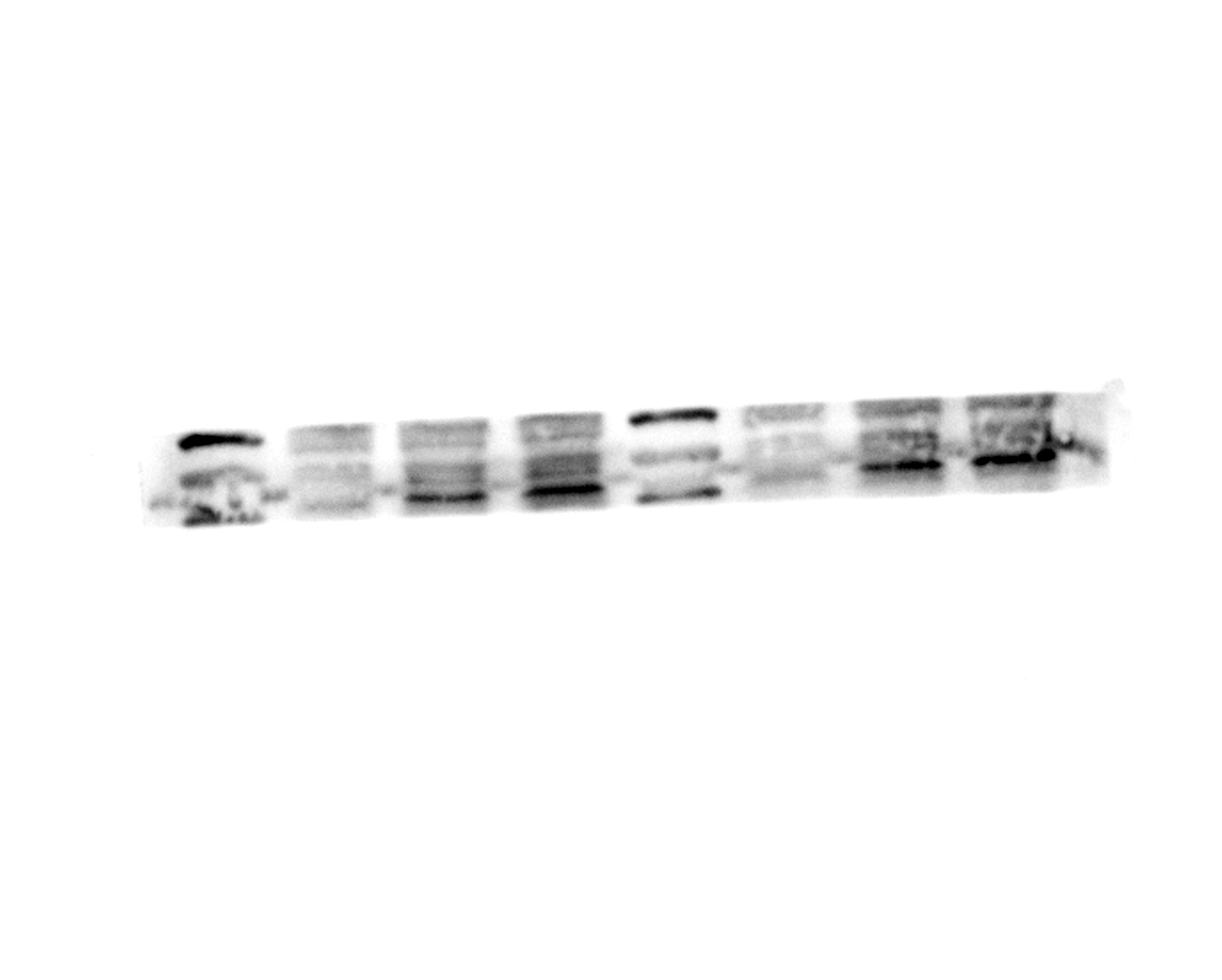

Supplement: Supplementary file 2 [file DataSheet1.zip › Supplementary file/Figure7c-IL-18.Tif]

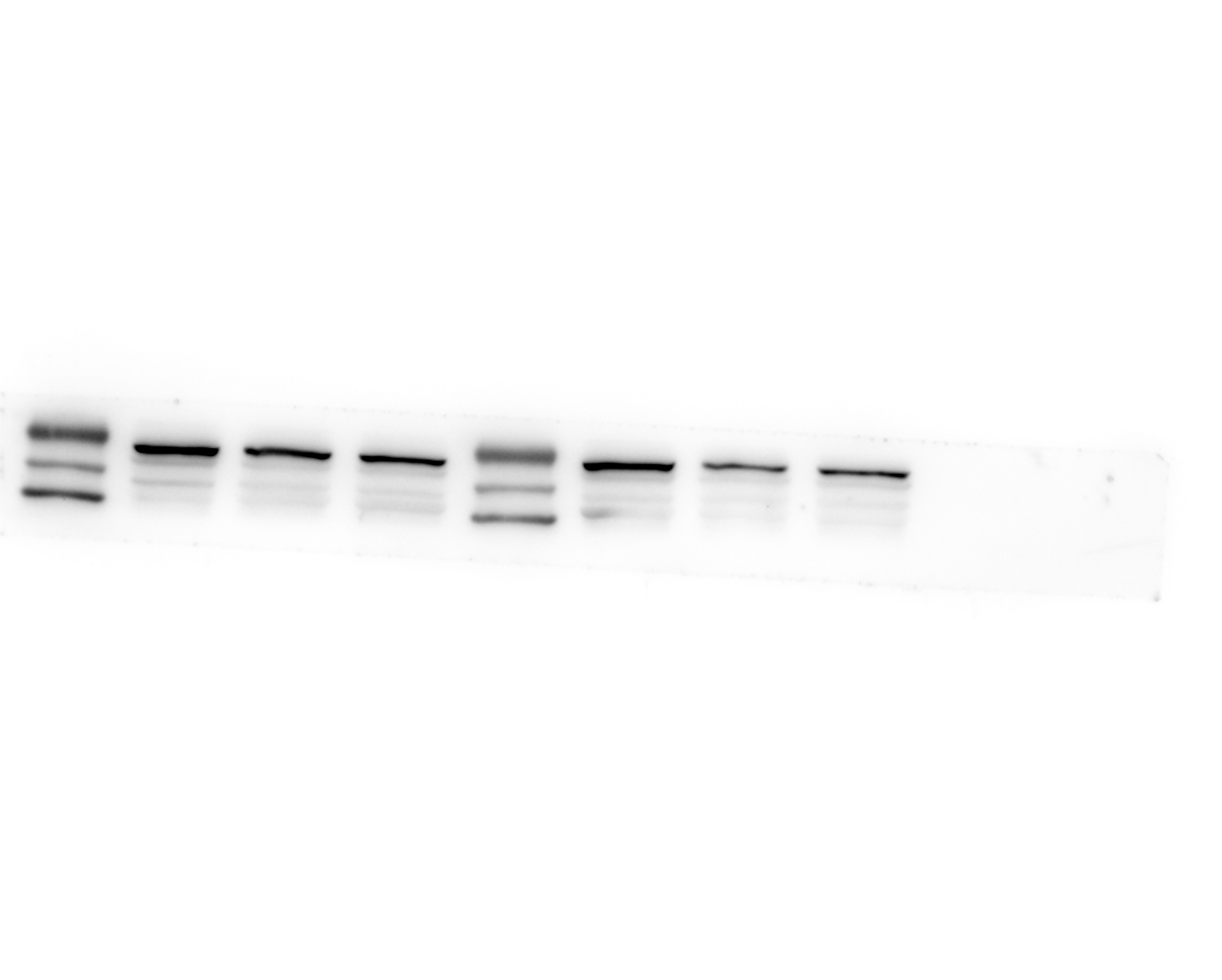

Supplement: Supplementary file 2 [file DataSheet1.zip › Supplementary file/Figure6a-IRF7-2.tif]

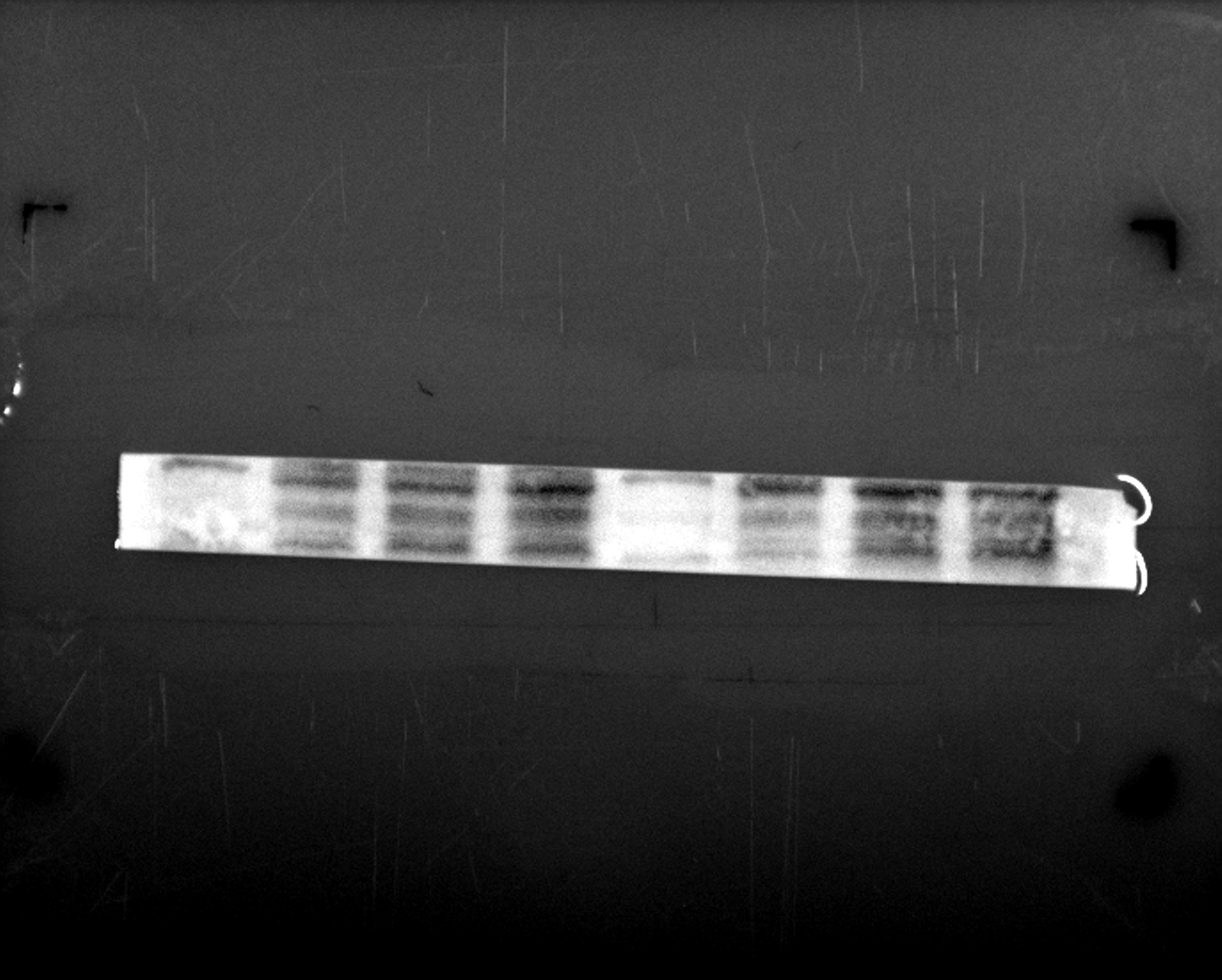

Supplement: Supplementary file 2 [file DataSheet1.zip › Supplementary file/Figure7c-IL-1b-merge.tif]

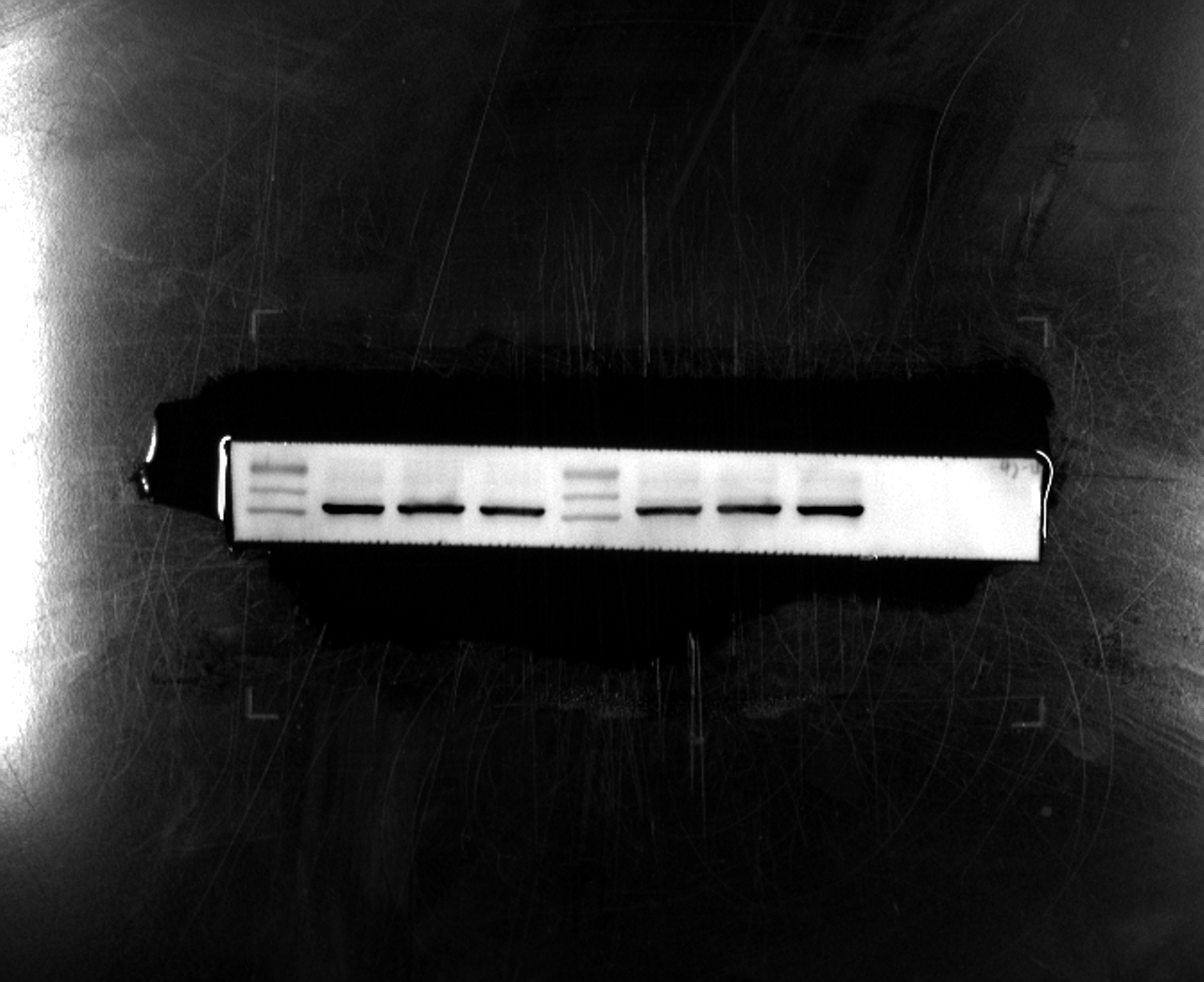

Supplement: Supplementary file 2 [file DataSheet1.zip › Supplementary file/Figure6a-bactin-2-merge.Tif]

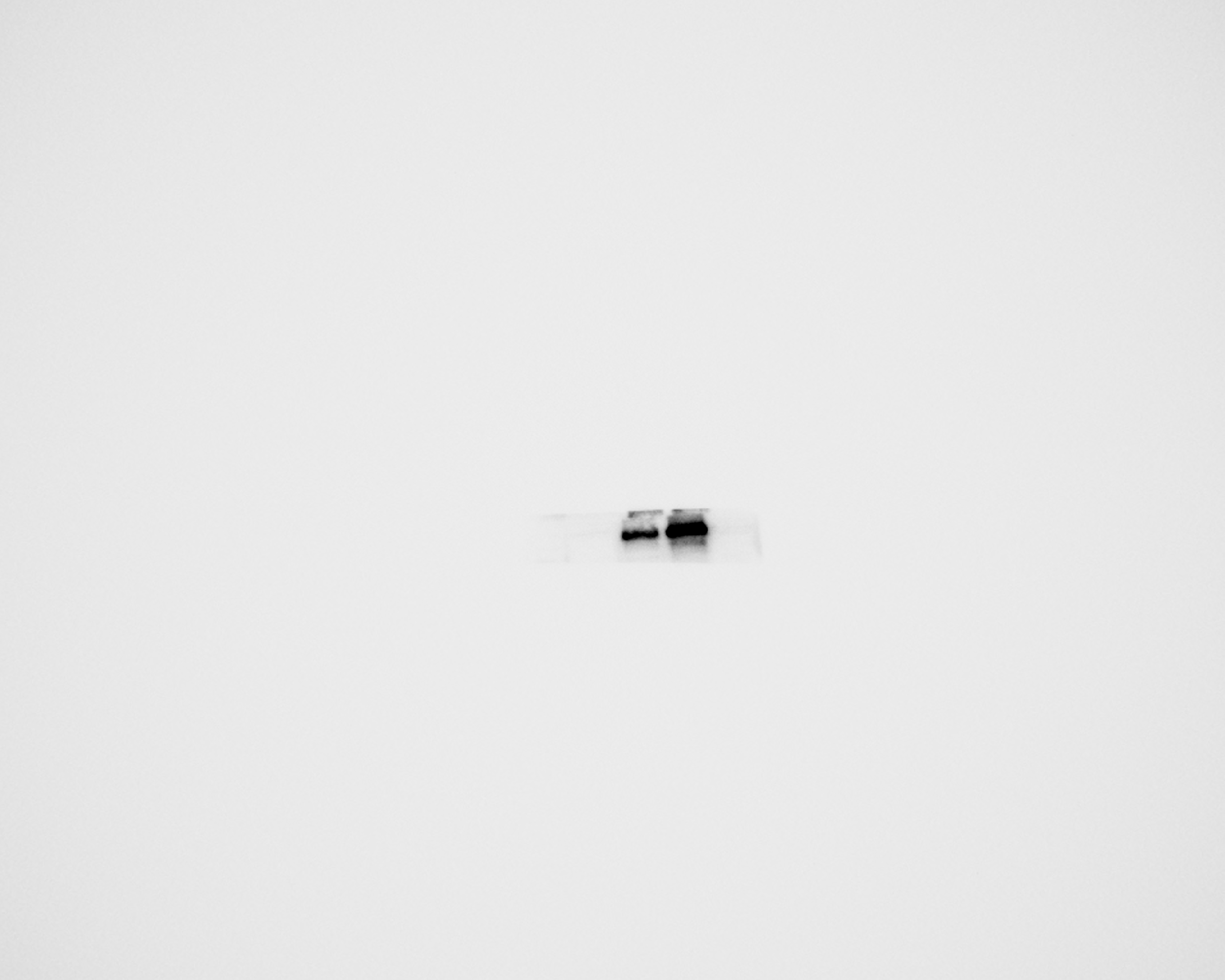

Supplement: Supplementary file 2 [file DataSheet1.zip › Supplementary file/Figure8g-IRF7.tif]

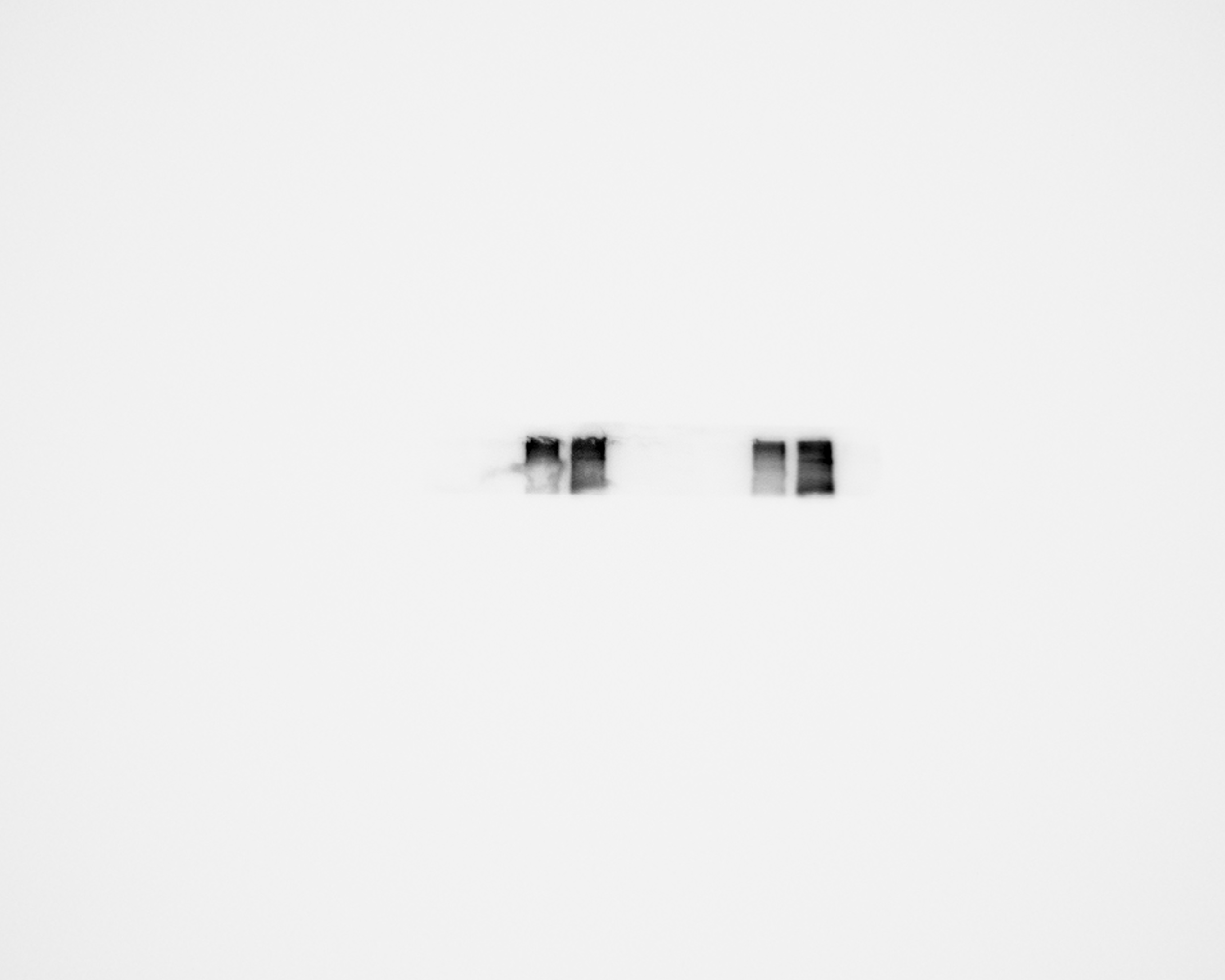

Supplement: Supplementary file 2 [file DataSheet1.zip › Supplementary file/Figure8g-NLRP3.tif]

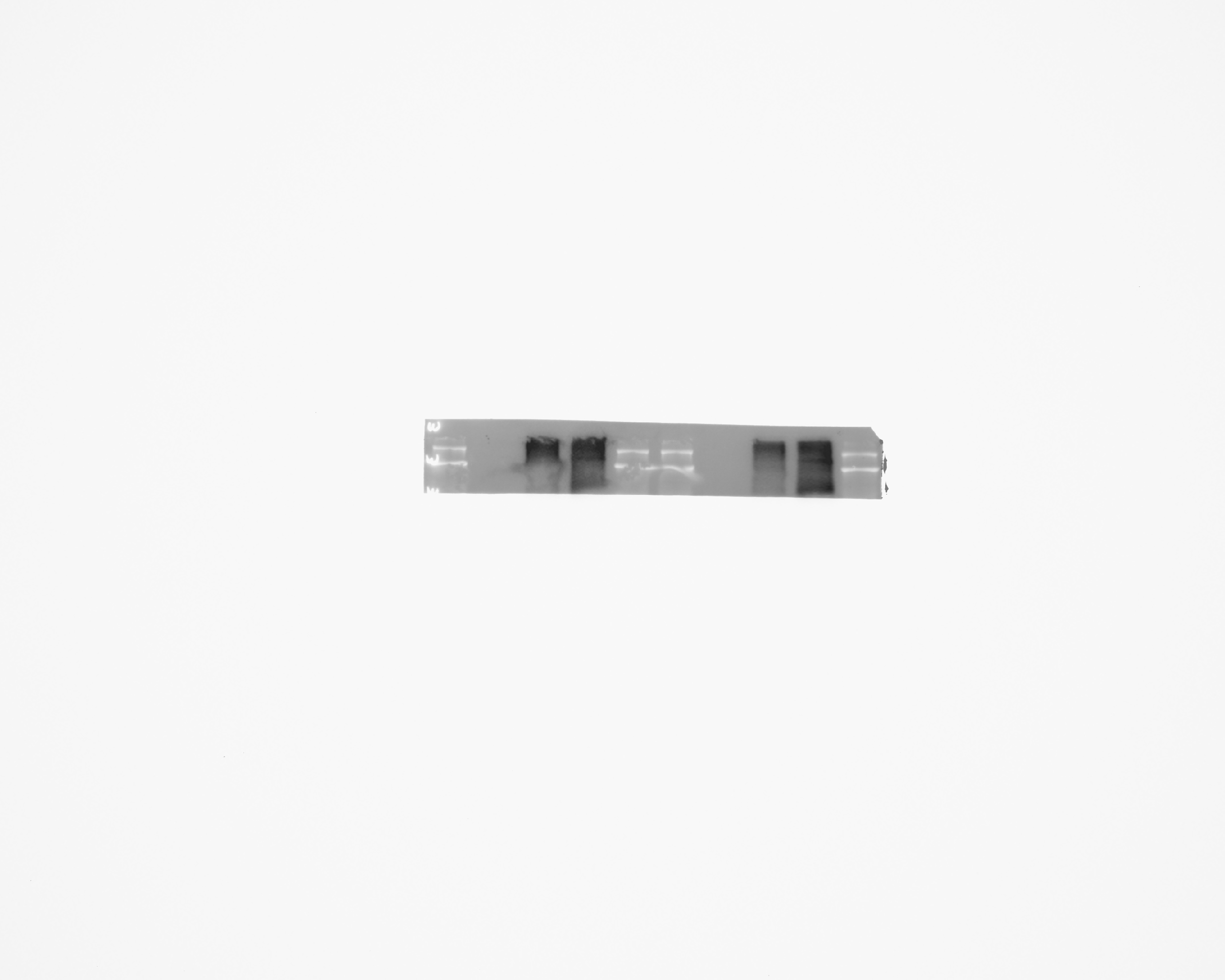

Supplement: Supplementary file 2 [file DataSheet1.zip › Supplementary file/Figure8g-NLPR3-merge1.tif]

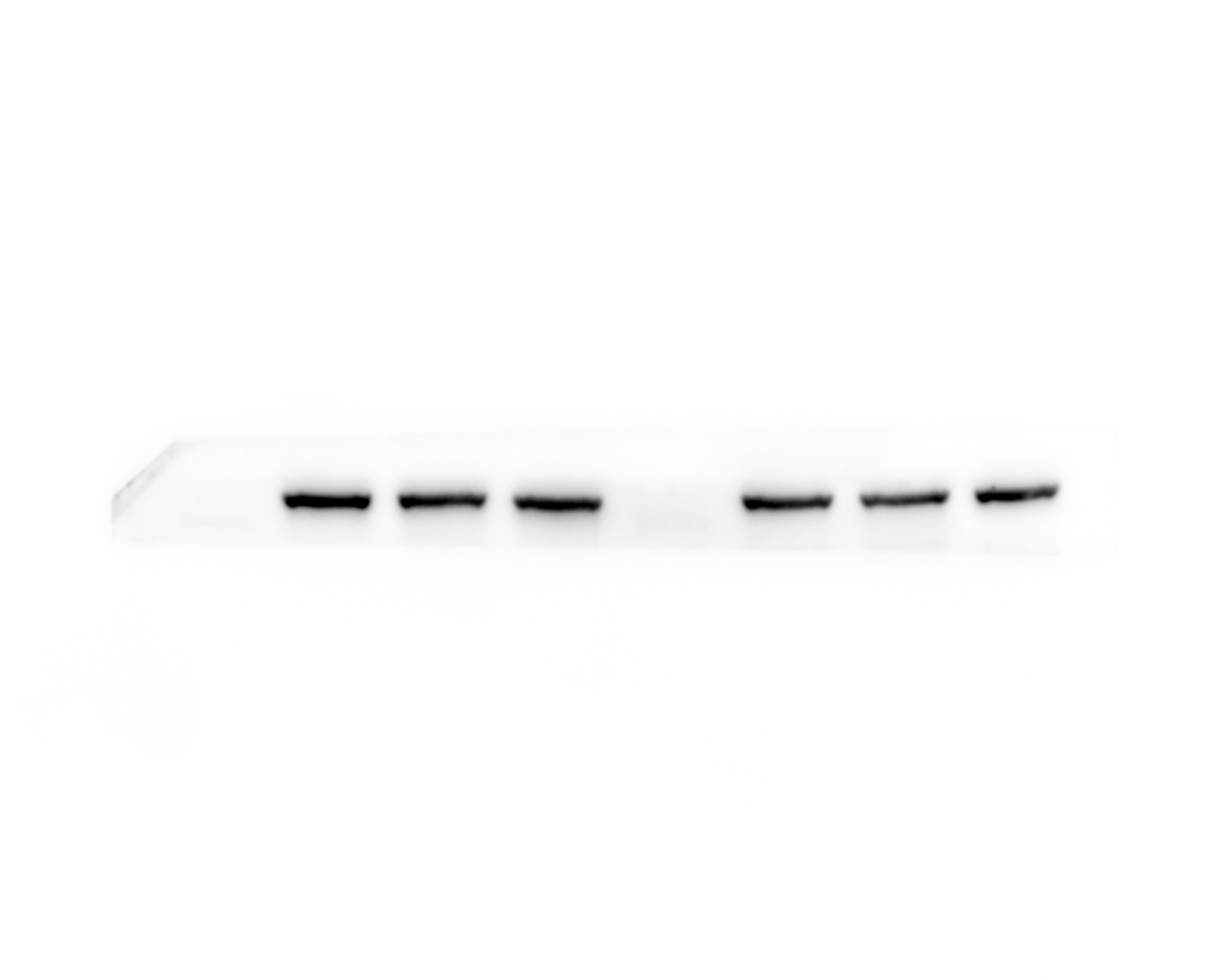

Supplement: Supplementary file 2 [file DataSheet1.zip › Supplementary file/Figure7c-bactin.Tif]

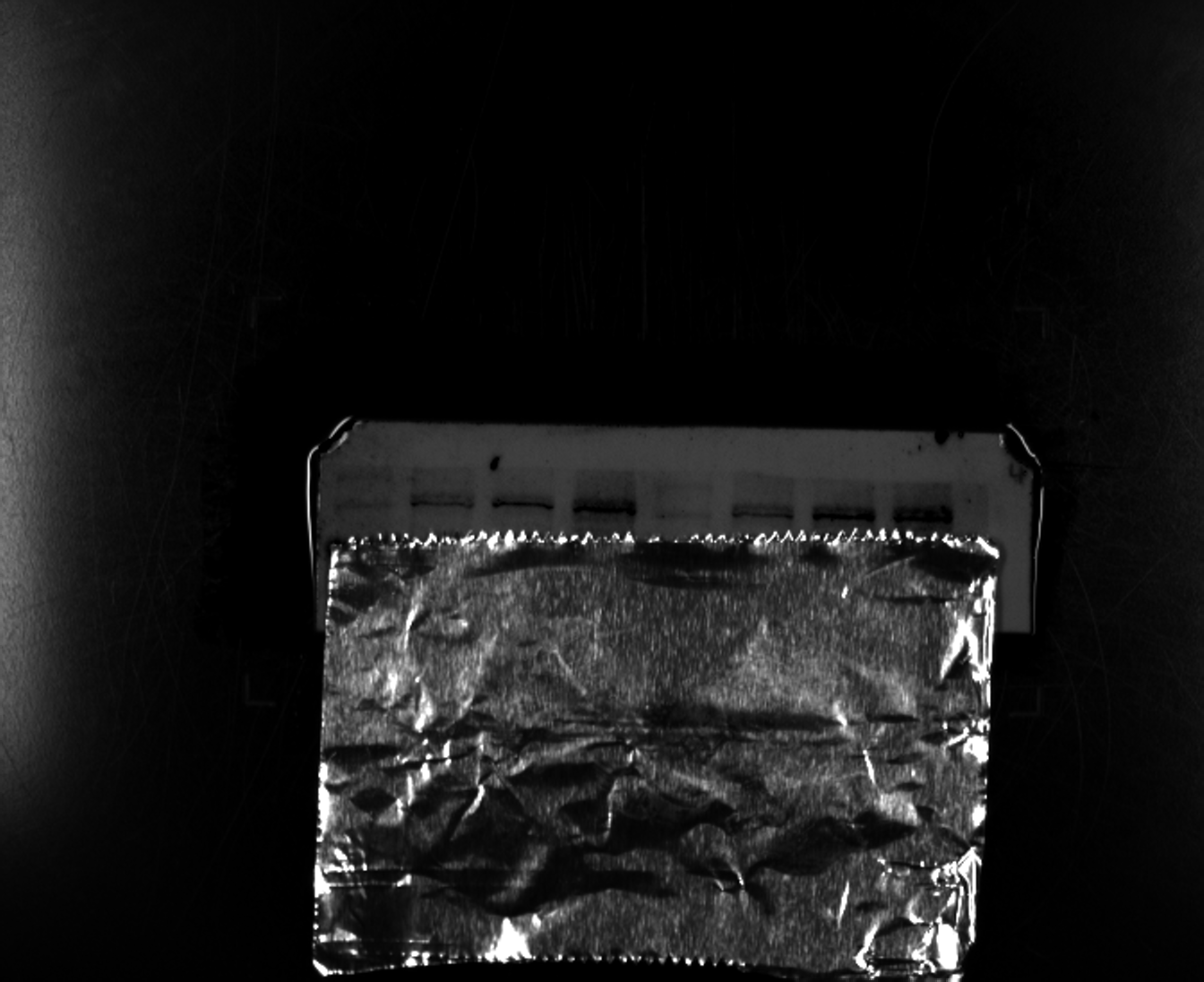

Supplement: Supplementary file 2 [file DataSheet1.zip › Supplementary file/Figure7c-NLRP3-merge.Tif]

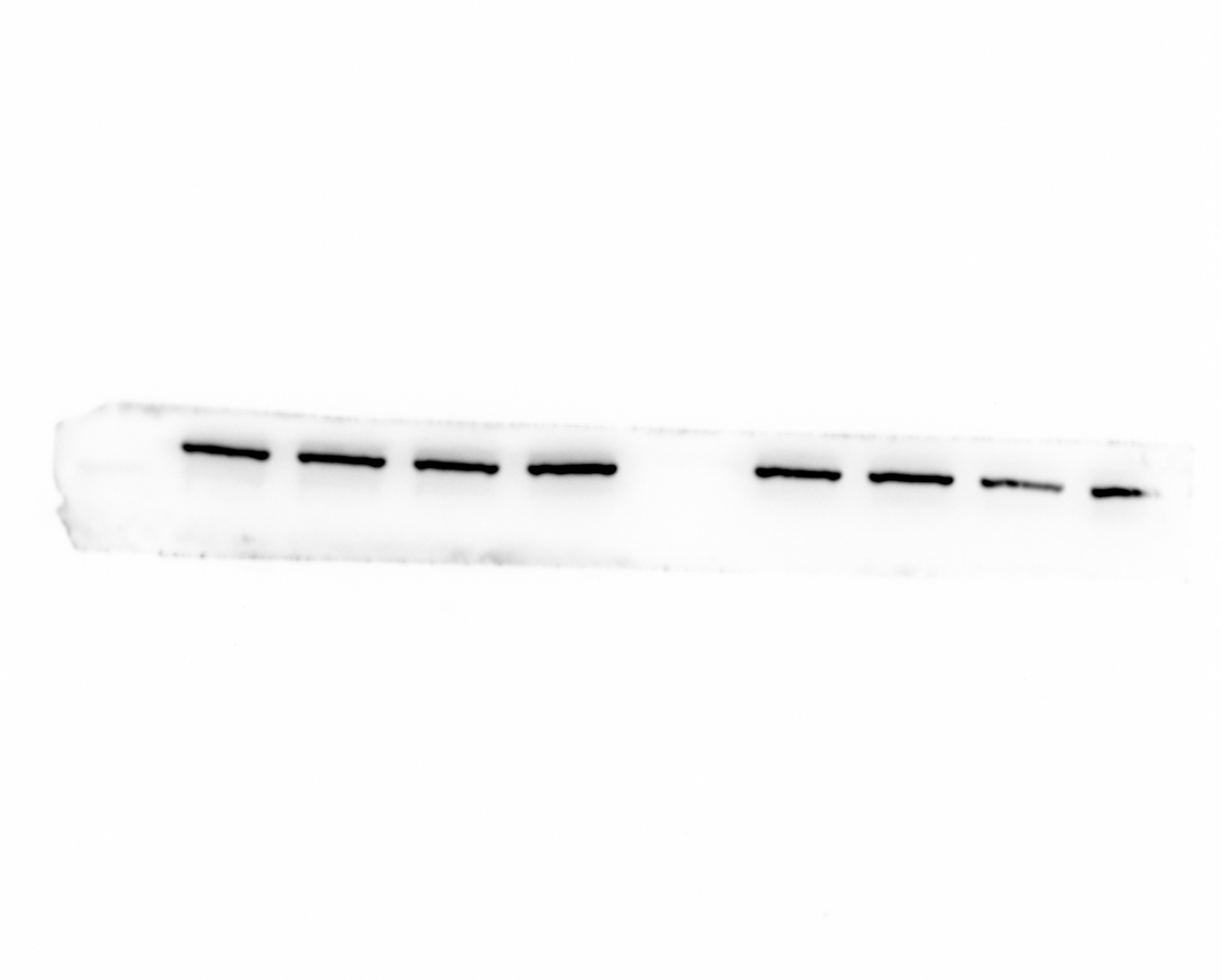

Supplement: Supplementary file 2 [file DataSheet1.zip › Supplementary file/Figure5d-bacitn.Tif]

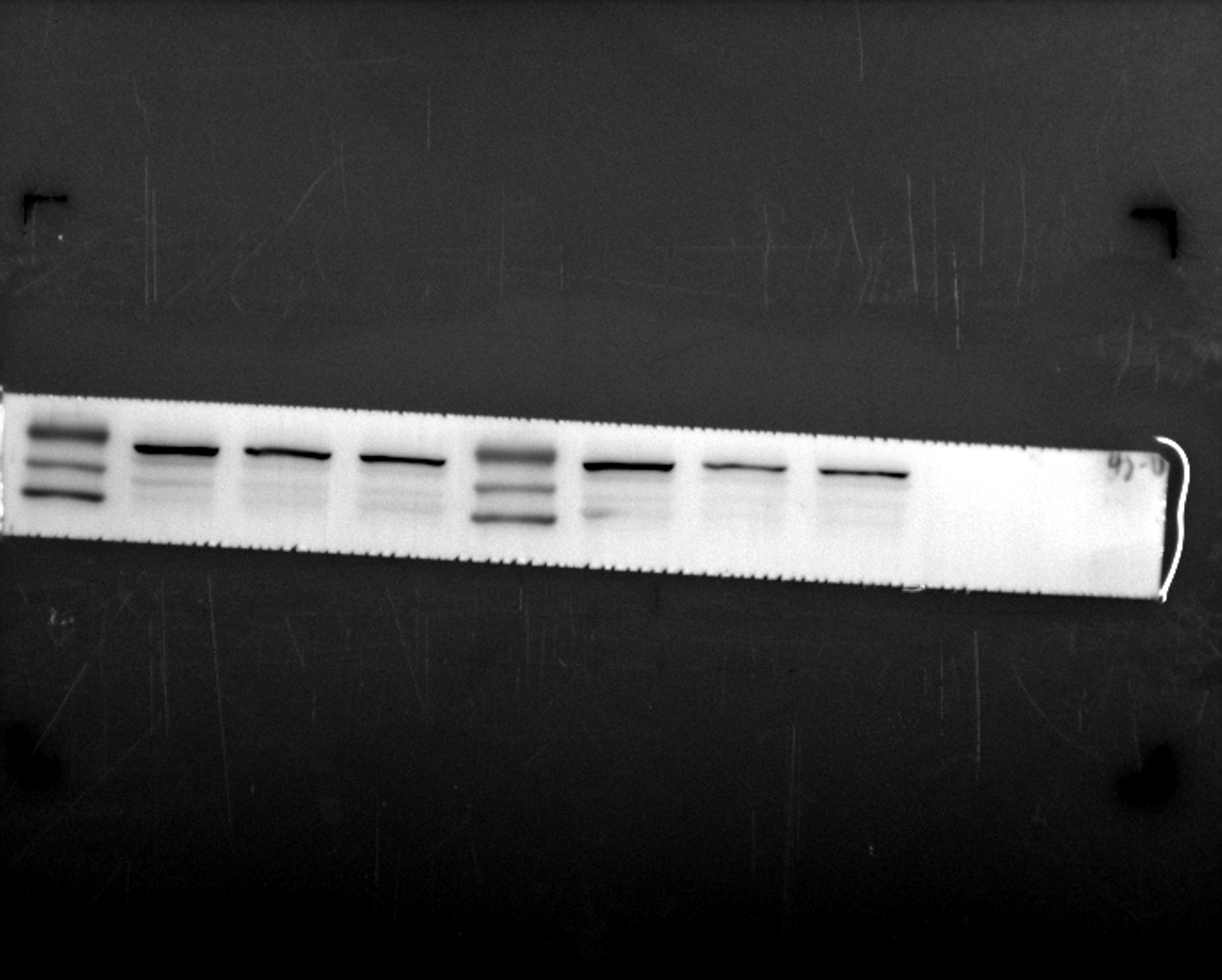

Supplement: Supplementary file 2 [file DataSheet1.zip › Supplementary file/Figure6a-IRF7-2-merge.tif]

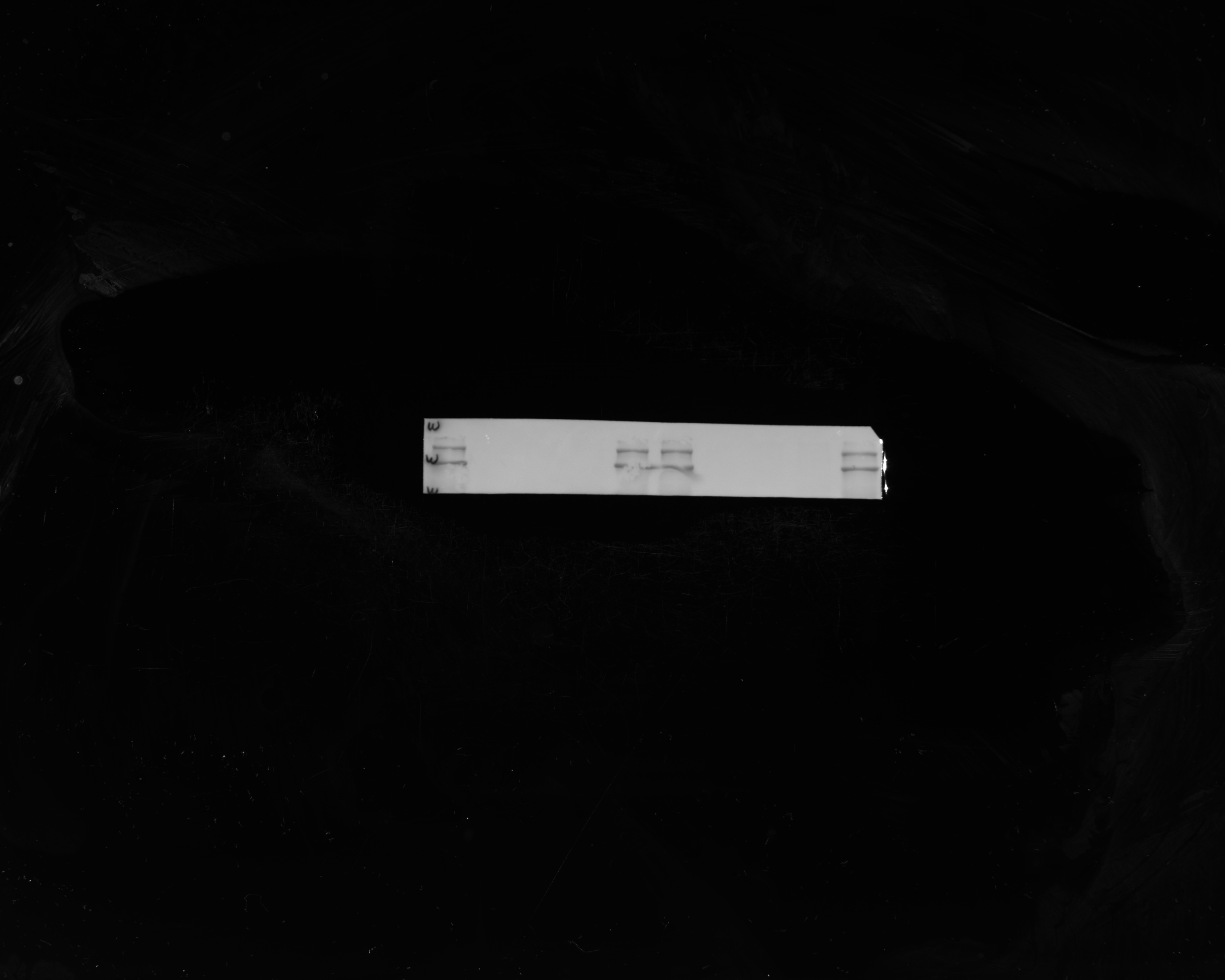

Supplement: Supplementary file 2 [file DataSheet1.zip › Supplementary file/Figure8g-NLRP3-merge2.tif]

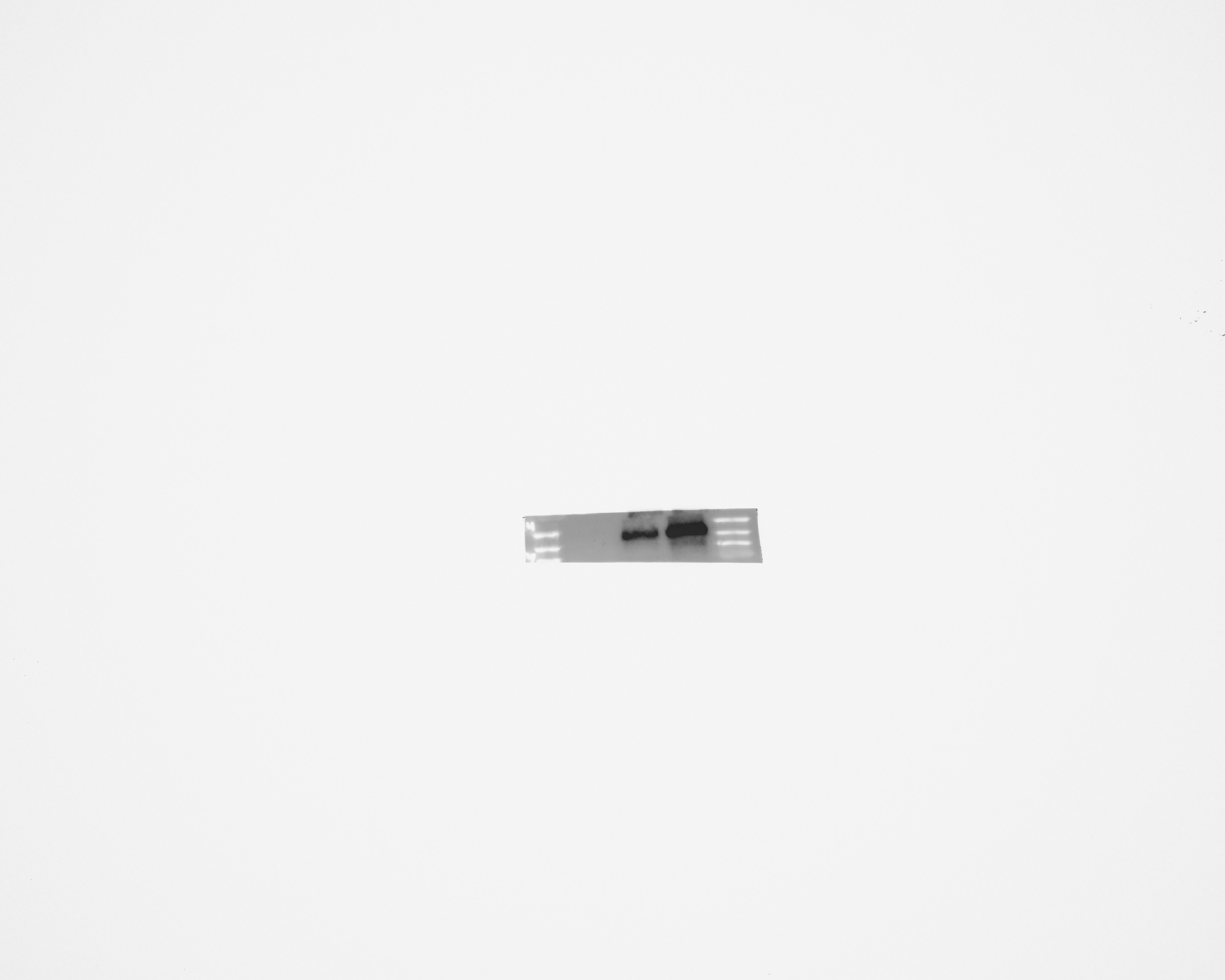

Supplement: Supplementary file 2 [file DataSheet1.zip › Supplementary file/Figure8g-IRF7-merge.tif]

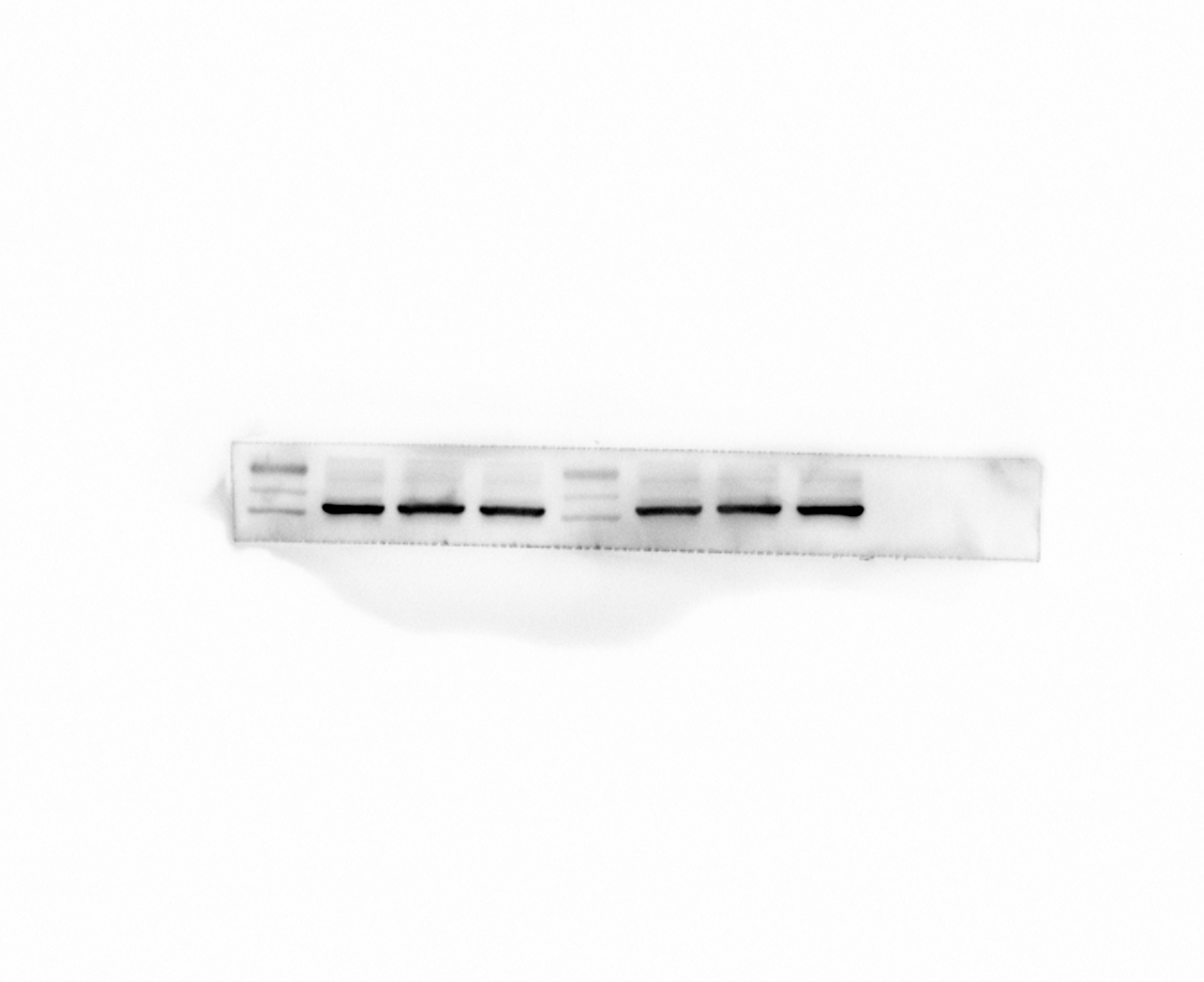

Supplement: Supplementary file 2 [file DataSheet1.zip › Supplementary file/Figure6a-bactin-2.Tif]

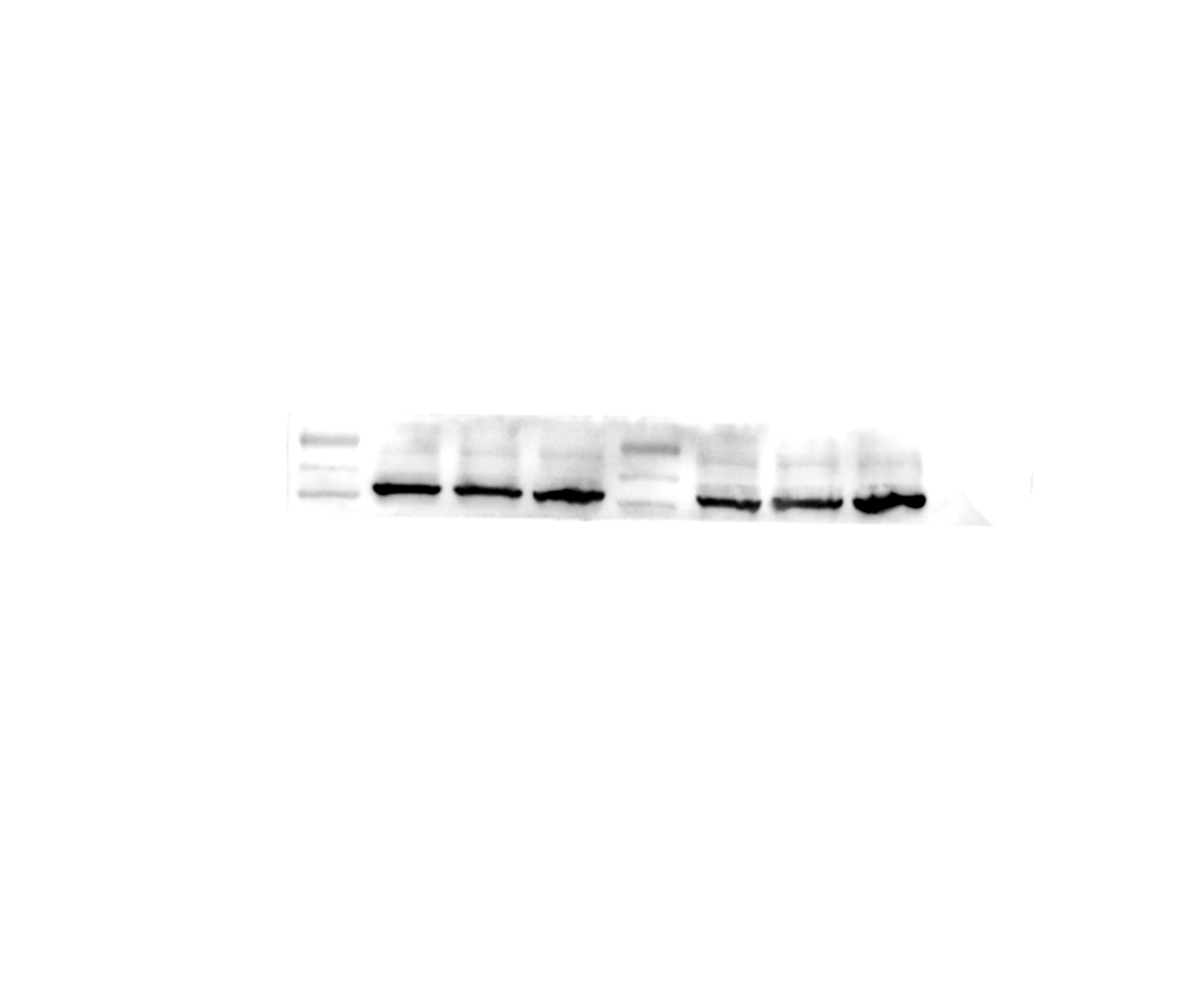

Supplement: Supplementary file 2 [file DataSheet1.zip › Supplementary file/Figure6a-bactin-1.Tif]

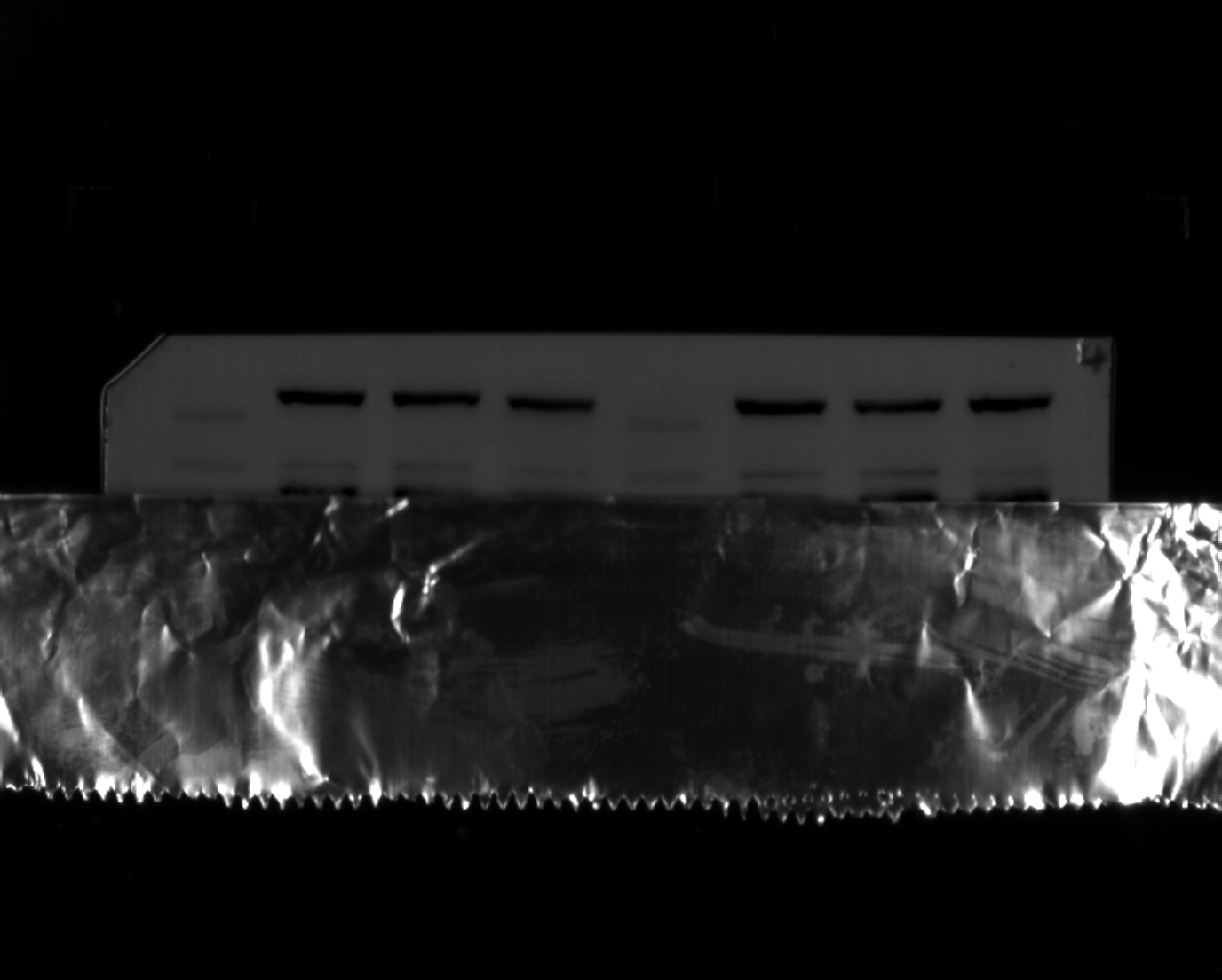

Supplement: Supplementary file 2 [file DataSheet1.zip › Supplementary file/Figure7c-bactin-merge.Tif]

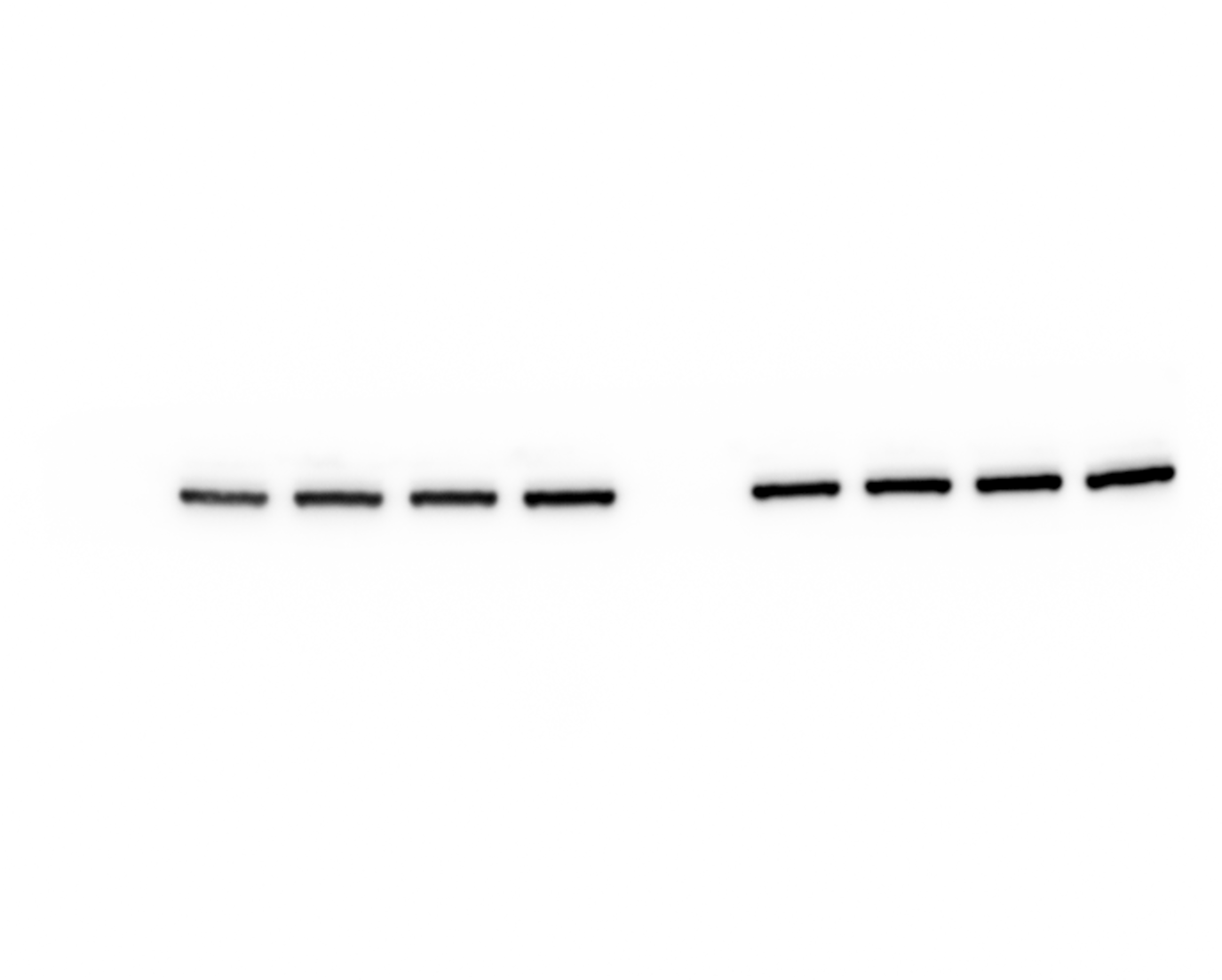

Supplement: Supplementary file 2 [file DataSheet1.zip › Supplementary file/Figure5d-IRF7.Tif]

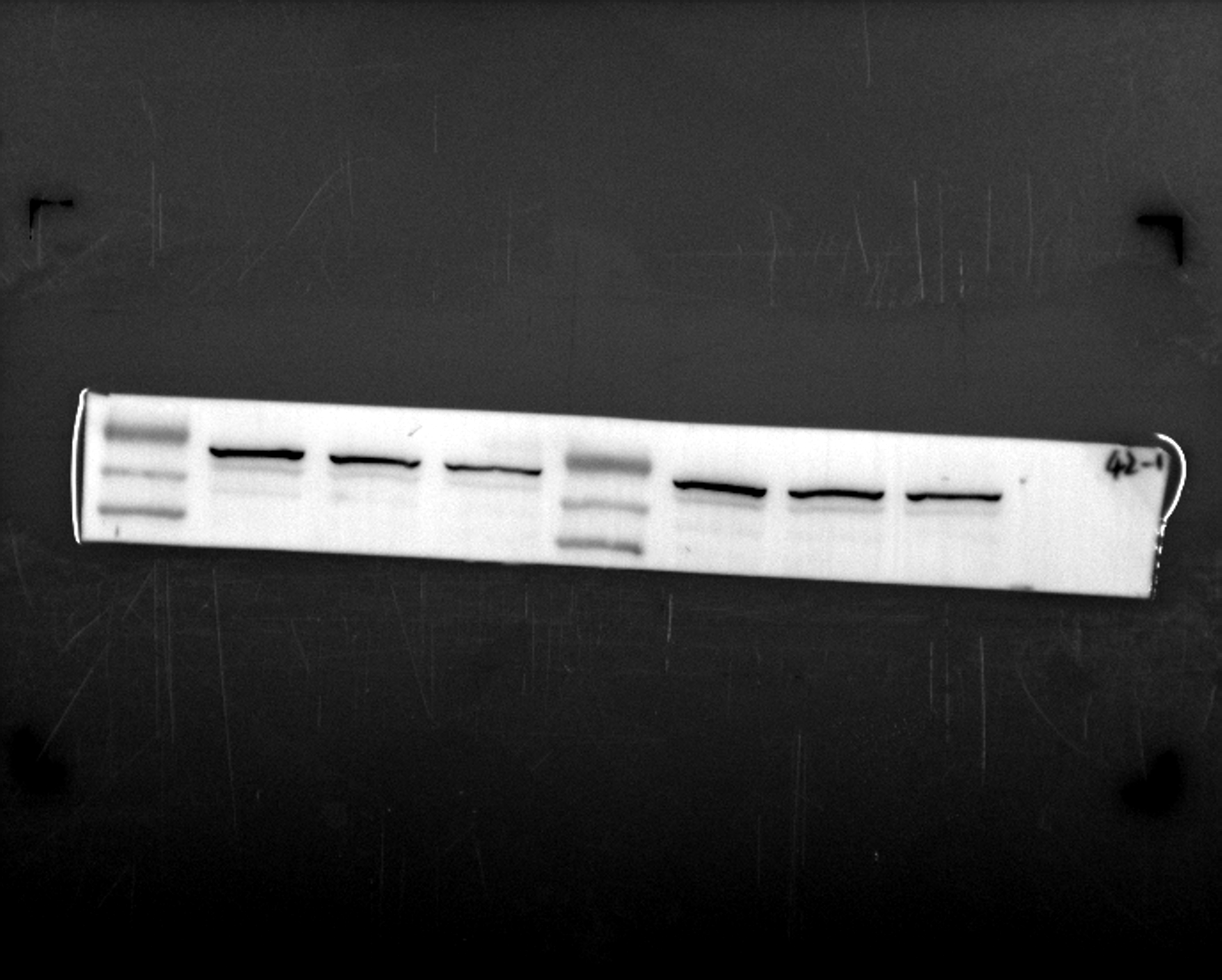

Supplement: Supplementary file 2 [file DataSheet1.zip › Supplementary file/Figure6a-IRF7-1-merge.tif]

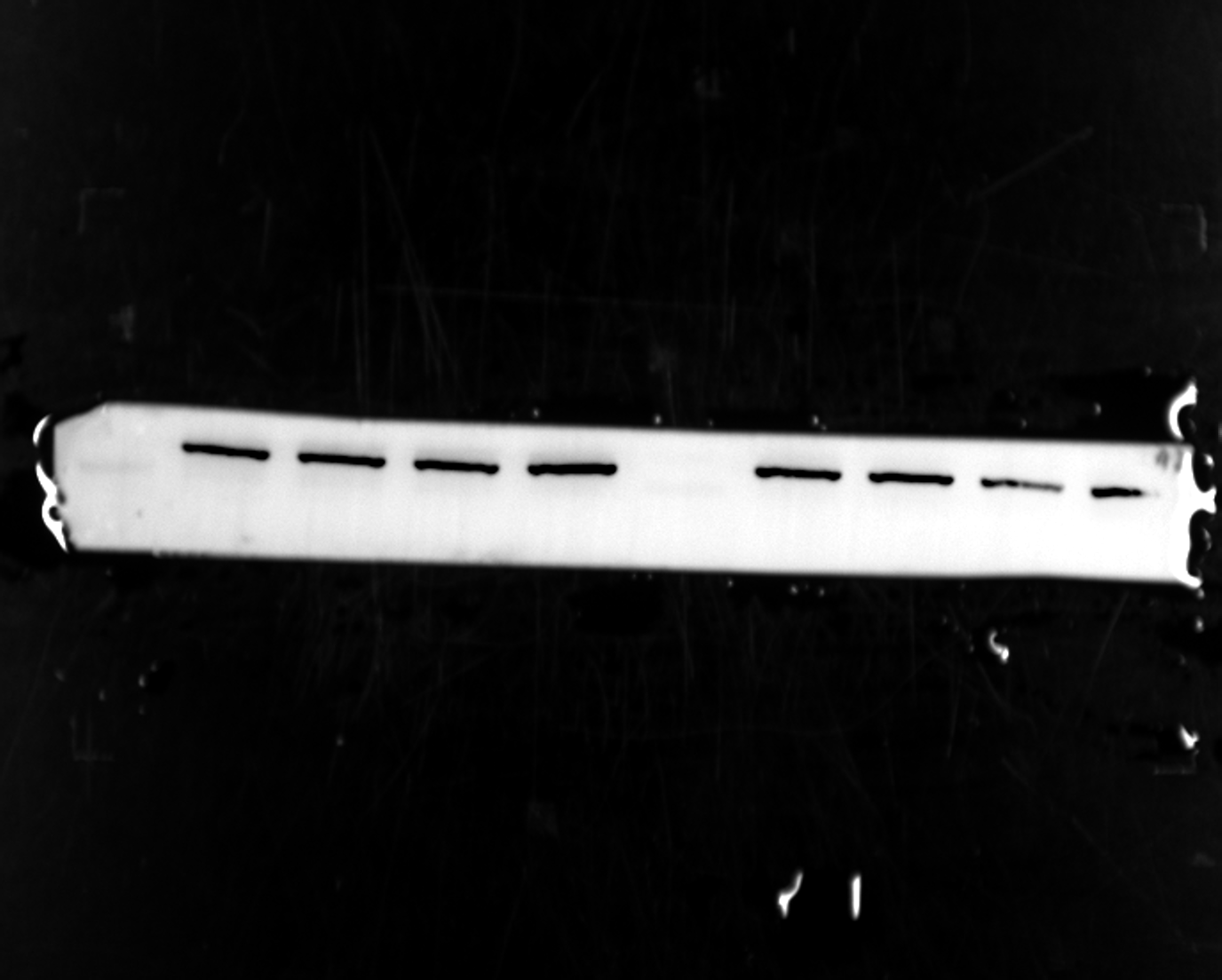

Supplement: Supplementary file 2 [file DataSheet1.zip › Supplementary file/Figure5d-bacitin-merge.Tif]

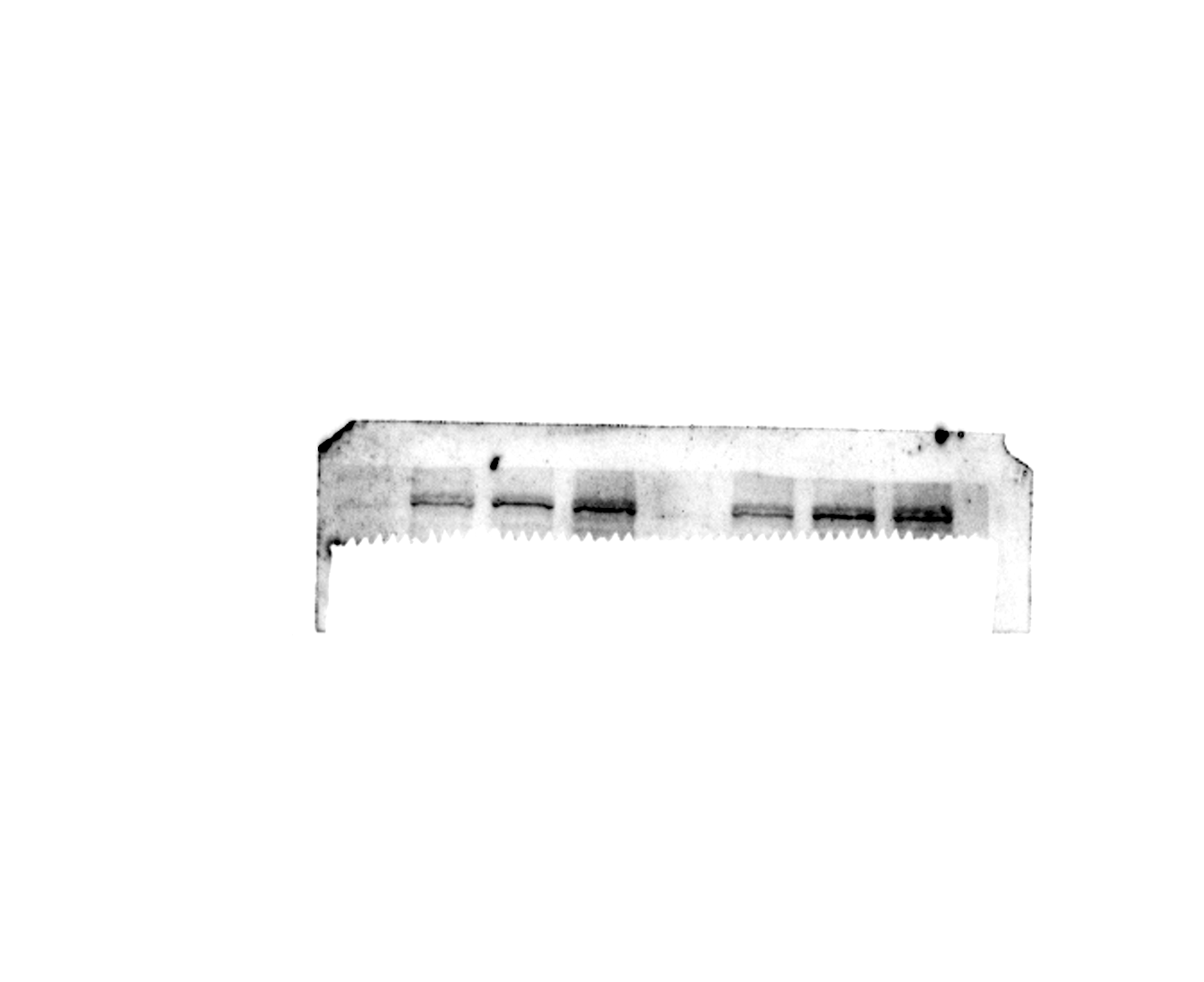

Supplement: Supplementary file 2 [file DataSheet1.zip › Supplementary file/Figure7c-NLRP3.Tif]

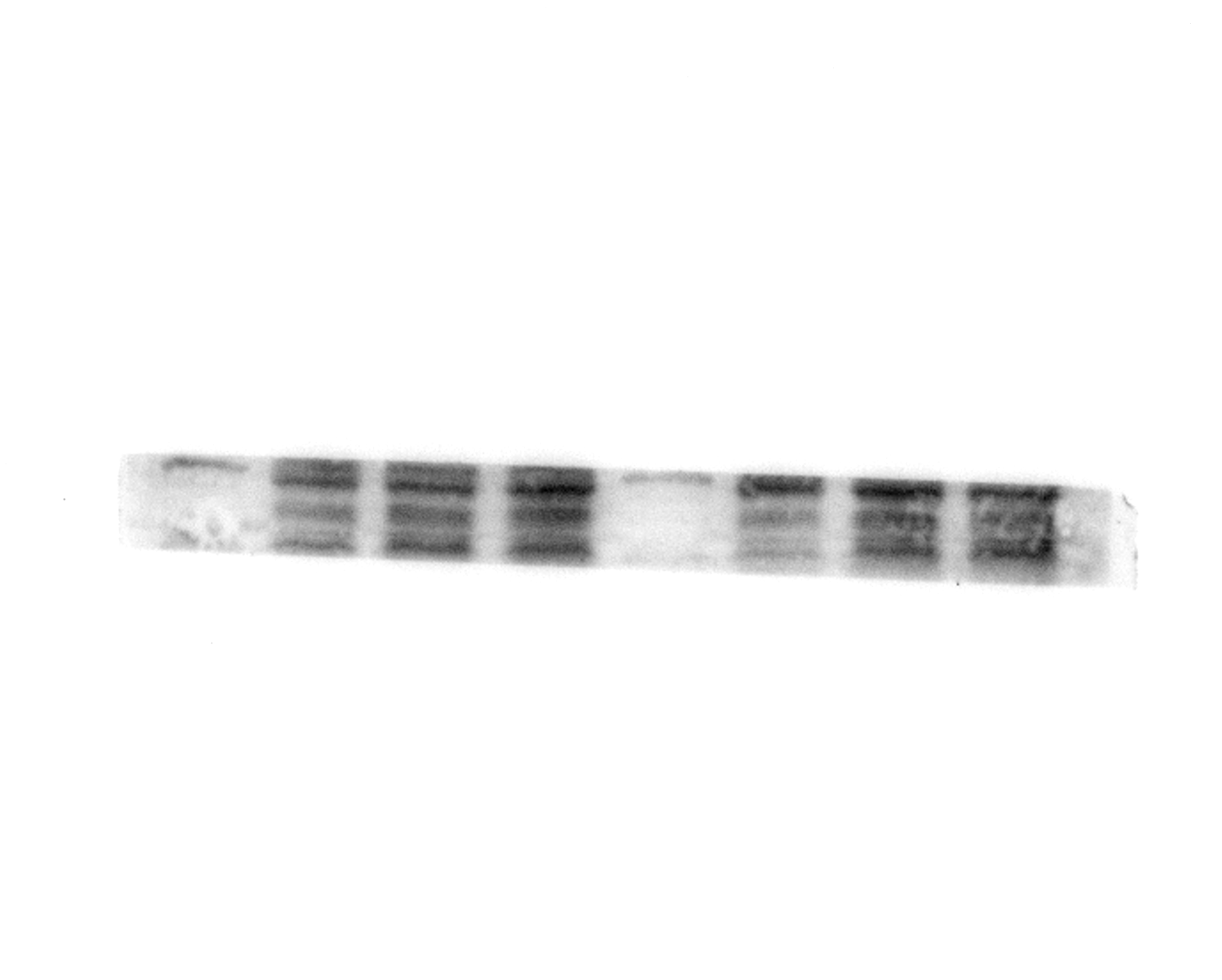

Supplement: Supplementary file 2 [file DataSheet1.zip › Supplementary file/Figure7c-IL-1b.tif]

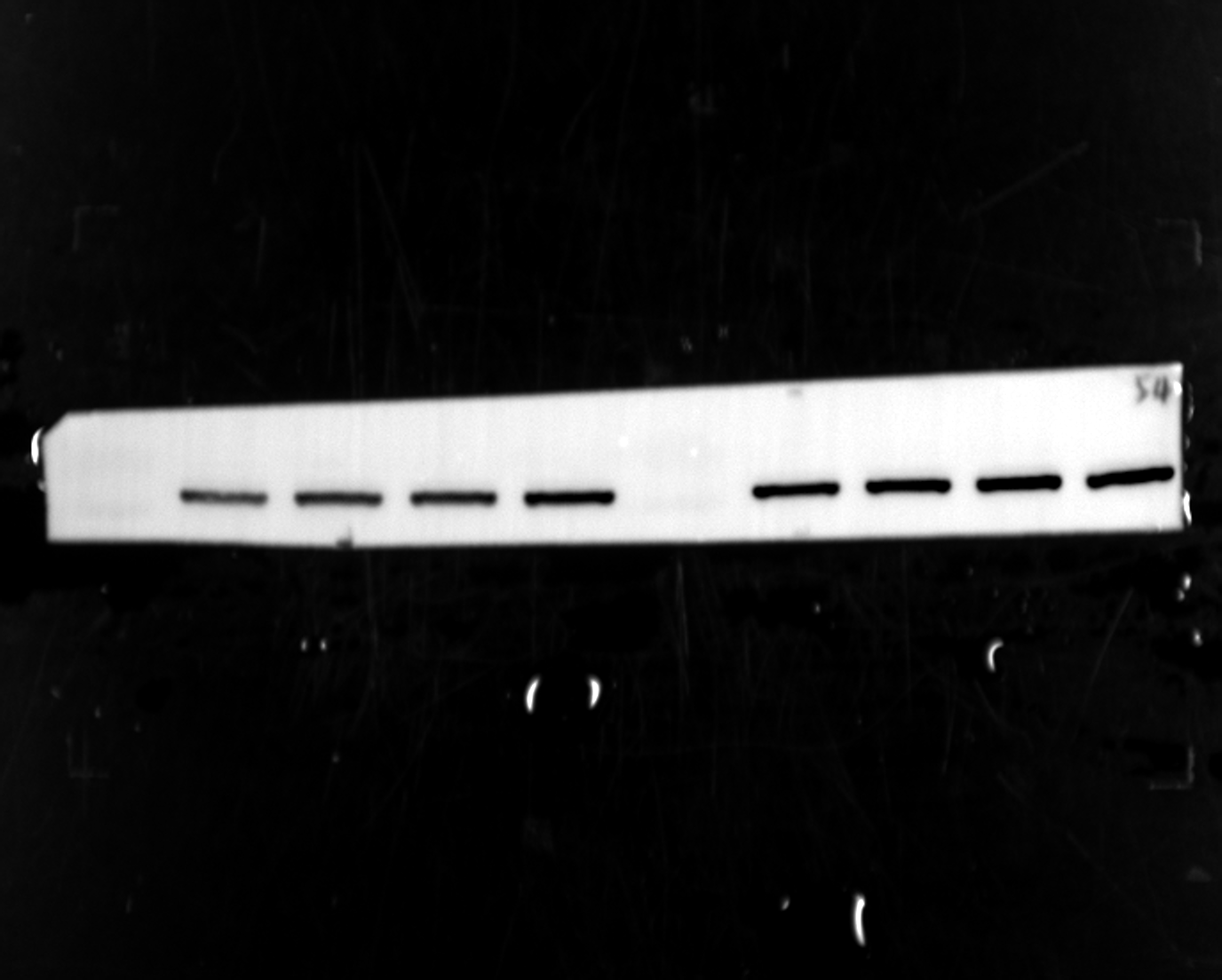

Supplement: Supplementary file 2 [file DataSheet1.zip › Supplementary file/Figure5d-IRF7-merge.Tif]

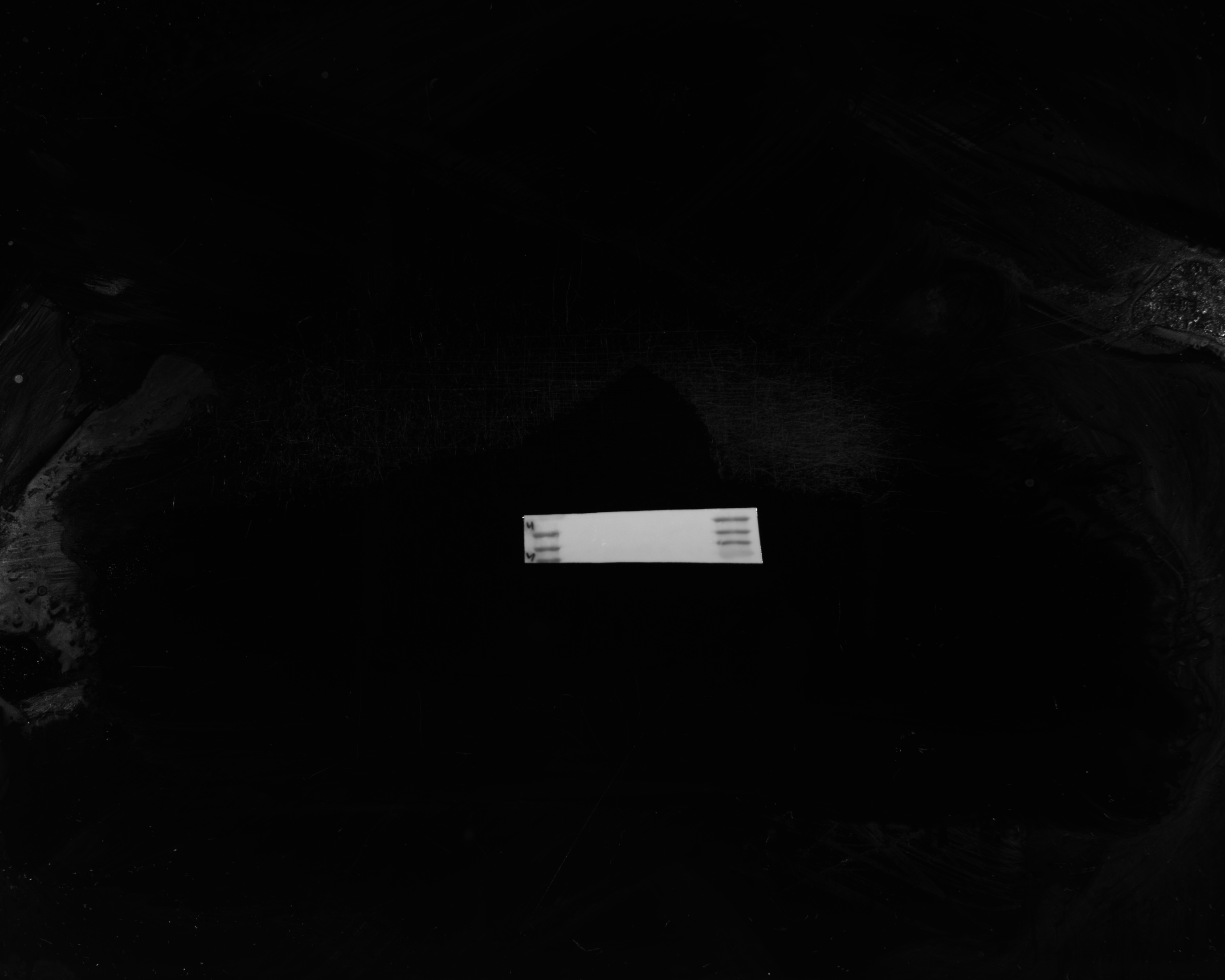

Supplement: Supplementary file 2 [file DataSheet1.zip › Supplementary file/Figure8g-IRF7-merge2.tif]

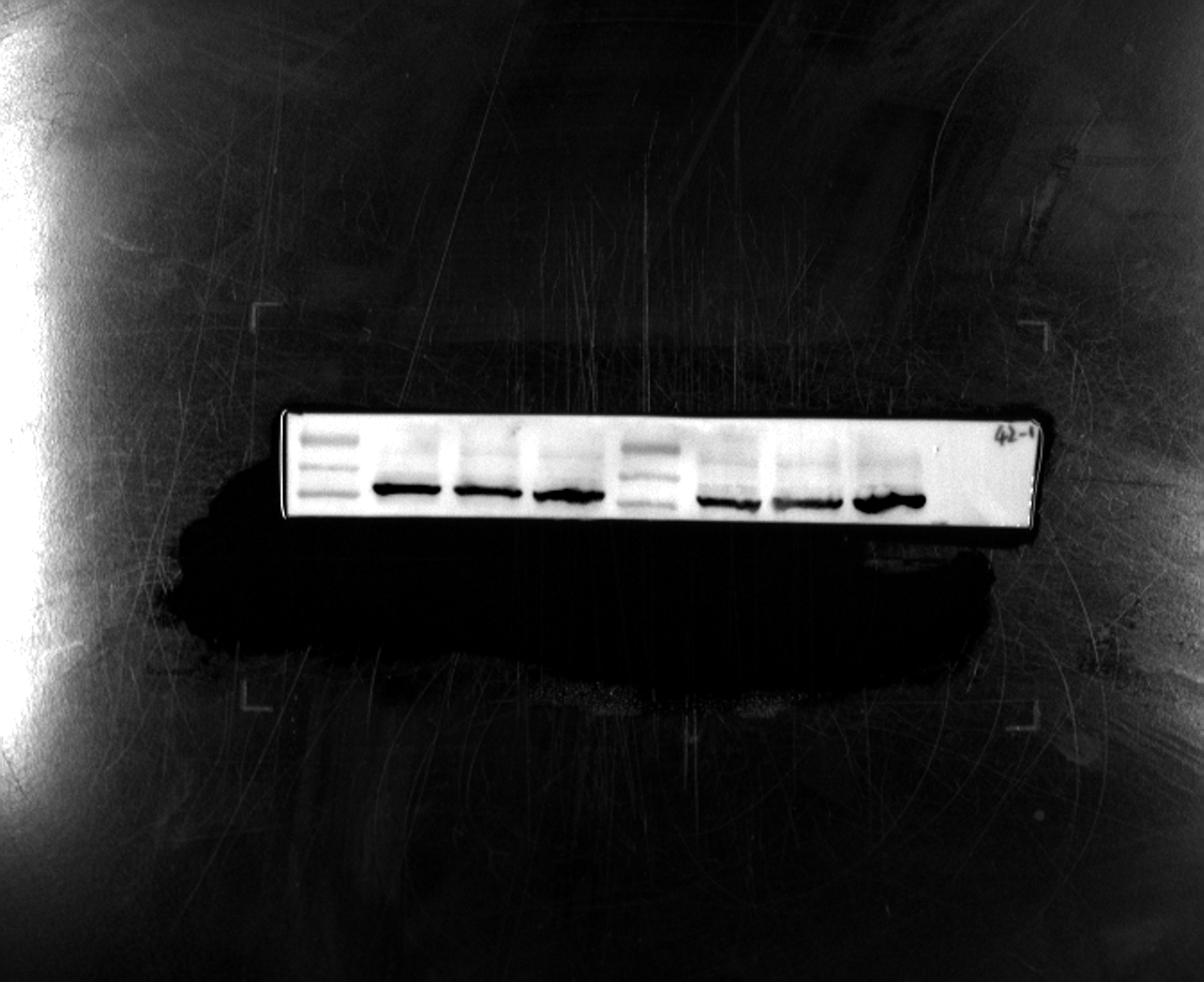

Supplement: Supplementary file 2 [file DataSheet1.zip › Supplementary file/Figure6a-bactin-1-merge.Tif]
